# Oncogene-induced reactive oxygen species fuel hyperproliferation and DNA damage response activation

**DOI:** 10.1038/cdd.2014.16

**Published:** 2014-02-28

**Authors:** M Ogrunc, R Di Micco, M Liontos, L Bombardelli, M Mione, M Fumagalli, V G Gorgoulis, F d'Adda di Fagagna

**Affiliations:** 1 IFOM Foundation, The FIRC Institute of Molecular Oncology Foundation, via Adamello 16, Milan, Italy; 2 Molecular Carcinogenesis Group, Department of Histology and Embryology, School of Medicine, University of Athens, 75 Mikras Asias Street, Goudi, 11527 Athens, Greece; 3 Basic Science II Center, Biomedical Research Foundation of the Academy of Athens, 4 Soranou Ephessiou Street, 11527 Athens, Greece; 4 Istituto di Genetica Molecolare, Consiglio Nazionale delle Ricerche, via Abbiategrasso 207, Pavia, Italy

**Keywords:** DNA damage response (DDR), oncogene-induced cellular senescence, pancreatic cancer, reactive oxygen species (ROS), zebrafish

## Abstract

Oncogene-induced reactive oxygen species (ROS) have been proposed to be signaling molecules that mediate proliferative cues. However, ROS may also cause DNA damage and proliferative arrest. How these apparently opposite roles can be reconciled, especially in the context of oncogene-induced cellular senescence, which is associated both with aberrant mitogenic signaling and DNA damage response (DDR)-mediated arrest, is unclear. Here, we show that ROS are indeed mitogenic signaling molecules that fuel oncogene-driven aberrant cell proliferation. However, by their very same ability to mediate cell hyperproliferation, ROS eventually cause DDR activation. We also show that oncogenic Ras-induced ROS are produced in a Rac1 and NADPH oxidase (Nox4)-dependent manner. In addition, we show that Ras-induced ROS can be detected and modulated in a living transparent animal: the zebrafish. Finally, in cancer we show that Nox4 is increased in both human tumors and a mouse model of pancreatic cancer and specific Nox4 small-molecule inhibitors act synergistically with existing chemotherapic agents.

Oncogene activation is a key step in cellular transformation and maintenance of cancer.^1^ However, upon activation, several oncogenes have been reported to cause a proliferative arrest, termed cellular senescence, which has cell-intrinsic tumor suppressive functions^2, 3, 4^ observed both *in vitro* and *in vivo*.^5, 6, 7, 8, 9^ Oncogene-induced cellular senescence (OIS) was first observed in normal fibroblasts upon ectopic expression of H-RasV12,^10^ and this remains the cellular system most frequently used. We have previously demonstrated that oncogene activation in normal cells leads to an initial hyperproliferative phase, which is transient and inevitably ends with the permanent establishment of cellular senescence.^11^ Rampant oncogene-induced hyperproliferation is transient because it is intrinsically associated with a still ill-characterized alteration of the DNA replication process, which causes the consequent activation of the checkpoint functions of the DNA damage response (DDR).^11, 12^ Thus, DDR precedes senescence establishment. DDR inactivation allows OIS bypass and thus the proliferation of oncogene-expressing cells and their transformation.^11, 12^ DDR activation has been reported in human tumor samples, especially in the early phases of carcinogenesis.^13, 14^

The increase in the accumulation of reactive oxygen species (ROS) in cells expressing an oncogene has been widely reported.^15, 16^ ROS may have a variety of functions in a cell with even apparently opposite outcomes. ROS may act as mitogenic signaling molecules and stimulate cell proliferation^15^ or be genotoxic and thus blunt proliferation by activating the DDR. Mitogenic ROS have been proposed to increase following the engagement of receptor tyrosine kinases and the activation of downstream signal transduction pathways, including Ras,^15, 17^ with NADPH oxidases (NOXs) being involved.^18, 19^ Under these conditions ROS act as signaling molecules mediating mitogenic cues and stimulate cell proliferation likely by inhibiting redox-sensitive phosphatases.^20^ ROS, however, can also generate DNA lesions by directly oxidizing its bases and the sugar backbone,^21^ and thus in this way activate the DDR checkpoint and arrest proliferation. The dual role of ROS is of particular relevance in understanding the mechanisms of establishment of OIS, as this involves the engagement of both mitogenic signal transduction pathways and antimitogenic (checkpoint enforcing) DDR signaling mechanisms.

At present, the scientific literature supports the notion that oncogenic Ras induces ROS accumulation^15^ in the cell and ROS scavenging reduces cellular senescence.^16^ However, the origins and most importantly how ROS have a causative role in OIS establishment are presently unclear.^22^

By studying the events occurring immediately following oncogene activation (H-RasV12) in human cells, we propose a model to reconcile the only apparently contradictory functions of ROS. We observed that oncogene-induced ROS have indeed mitogenic functions and drive the initial hyperproliferative phase that is, however, responsible for DDR activation. Thus, ROS are indeed genotoxic but, surprisingly, by virtue of their ability to mediate oncogene-driven augmented proliferation and aberrant DNA replication. This model of ROS-mediated OIS had never been proposed or demonstrated. We show that upon oncogene activation, if proliferation is not allowed, oncogene-induced ROS accumulate but they do not cause DDR activation. Rho-GTPases are known to be key downstream targets of Ras in the transduction of signals for particular biological outcomes.^23^ ROS production in many non-phagocyctic cells requires NOX enzyme activation.^18^ We observed that upon H-RasV12 activation, ROS accumulated in a RAC1- and NOX4-dependent manner. Importantly, we also show that the individual expression of these genes is sufficient to mimic H-RasV12 in terms of hyperproliferation induction, DDR activation and proliferative arrest.

We also show for the first time that oncogene-induced ROS accumulation can be detected in a living animal *Danio rerio* (zebrafish), a transparent vertebrate, and preventing ROS accumulation prevents the lethal phenotype associated with oncogene activation in this animal species.

Moreover, we studied these events in the context of cancer. We show that Nox4 expression is upregulated in human pancreatic tumor samples and in a genetically defined murine model of Ras-driven pancreatic cancer, where increased ROS levels can be visualized *in situ*. Finally, we report the exciting finding that specific NOX4 small-molecule inhibitors act synergistically with a first-line chemotherapeutic agent (gemcitabine) in a human pancreatic cancer cell line.

## Results

### ROS accumulation upon oncogene expression

The expression in normal human fibroblasts (NHF) of the activated and therefore oncogenic form of H-Ras (H-RasV12) leads to the establishment of cellular senescence.^10, 11^ Oncogene activation, cellular senescence and cell transformation are events associated with increased cellular ROS levels.^15, 16, 24^ We analyzed the contribution of ROS accumulation in the establishment of cellular senescence induced by H-RasV12 expression in NHF by the use of *N*-acetyl-cysteine (NAC), a broad-specificity ROS scavenger that acts by replenishing cellular glutathione;^25^ as a negative control, we used *N*-acetyl-alanine (NAA), a related but inactive compound. Intracellular ROS accumulation in the form of superoxide anions can be detected by fluorescence microscopy in living cells using the cell permeable ROS probe dihydroethidium (DHE)^26^ that, upon oxidation, accumulates in the nucleus and produces a red fluorescence. We observed that upon retroviral expression of H-RasV12 and selection in NHF, ROS accumulation as detected by DHE, is prevented by the presence of NAC, but not NAA, in the culture medium (Supplementary Figures S1A and B). ROS scavenging prevents OIS establishment, as demonstrated by ongoing cellular proliferation of oncogenic Ras-transduced cells as measured by increasing cell numbers and by BrdU incorporation assays (Supplementary Figures S1C and D). As control, NAA does not prevent OIS establishment and empty vector-infected cells proliferated vigorously in the presence of either NAC or NAA. These results are consistent with earlier reports^16^ and with a role of ROS as senescence-inducing molecules. However, given the dual role of mitogenic signaling molecules and genotoxic agents, these results do not provide a mechanism for senescence establishment by ROS.

**Figure 1:**
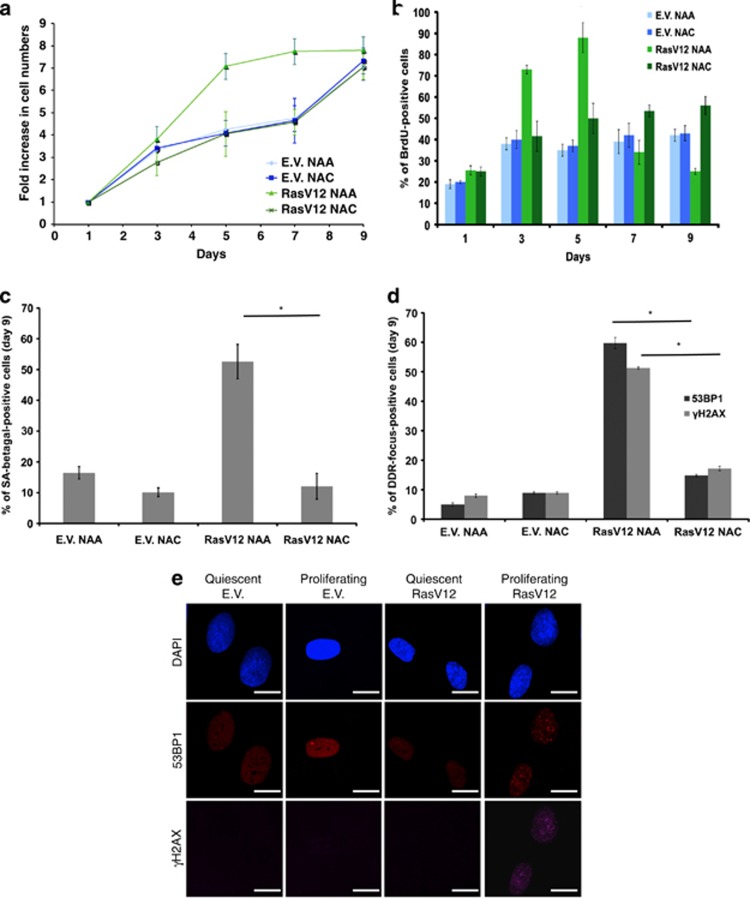
ROS scavenging prevents H-RasV12-induced intracellular ROS accumulation, cell hyperproliferation, DDR activation and OIS establishment. (**a**) NHFs were transduced with either an empty vector (E.V.) or a H-RasV12-expressing (Ras) retrovirus. Cell growth curves show that oncogenic Ras-induced hyperproliferation and OIS establishment can be prevented by the ROS scavenger NAC, but not its related inactive compound NAA. Proliferation is shown as the ratio of fold increase in cell numbers compared with day 1. Experimental scheme is shown in Supplementary Figure S1E. (**b**) BrdU incorporation rates, measured at the indicated time points of the growth curve shown in **a**, show that ROS scavenging prevents both oncogene-induced increased DNA replication rates and subsequent replicative arrest associated with OIS. BrdU incorporation rates of RasV12 NAA treatment are compared with RasV12 NAC treatment for day 3, 5 and 7. Error bars indicate S.E.M. (*n*≥ 3), and differences are statistically significant (*P-value < 0.01 and **P- value <0.05) throughout the figures where stated. (**c**) Senescence-associated beta galactosidase assays show that ROS scavenging prevents OIS establishment. The percentage of SA-beta galactosidase-positive cells in RasV12 NAA treatment is compared with RasV12 NAC treatment. Error bars indicate S.E.M. (*n* ≥ 3), and differences are statistically significant (*P-value < 0.01). (**d**) Quantification of DDR in the form of *γ*H2AX and 53BP1 foci of cells at the last time point of the growth curves as shown in **a**, indicates that ROS scavenging prevents DDR activation. The percentages of DDR in the form of *γ*H2AX and 53BP1 foci of cells are compared between RasV12 NAA and RasV12 NAC treatments. Error bars indicate S.E.M. (*n*≥ 3), and differences are statistically significant (*P-value < 0.01). (**e**) DDR in the form of *γ*H2AX and 53BP1 foci is activated upon RasV12 expression in proliferating, but not in quiescent cells. Scale bar: 10 *μ*m. (See also Supplementary Figure S1H)

Indeed, we have previously reported that OIS establishment is preceded by a strong proliferative burst immediately after oncogene expression by retroviral transduction, that is more evident in the absence of drug selection, and thus cannot be appreciated in Supplementary Figures S1C–E and^16^ where cell growth was studied only after drug selection. Hyperproliferation has a causal role in OIS establishment.^11^ We thus decided to test the potential role of oncogene-induced ROS also in the hyperproliferative phase, which precedes OIS establishment and can be best appreciated immediately after infection in the absence of drug selection (Supplementary Figure S1F). Strikingly, we observed that the presence of NAC, but not NAA, in the culture medium fully suppresses oncogene-driven cell hyperproliferation and allows cells to proliferate with a rate indistinguishable from empty vector-infected control cells, as determined both by cell number and BrdU incorporation rates (Figures 1a and b). Empty vector-transduced control cells behaved indistinguishably in the presence of NAA or NAC. Although Supplementary Figures S1C and D and the current literature support the notion that oncogene-induced ROS are molecules that reduce cell proliferation and cause cell senescence, these unexpected data indicate that ROS are essentially mitogenic molecules that cause senescence by first fueling hyperproliferation that is intrinsically genotoxic and by doing so they eventually cause the proliferative arrest known as cellular senescence.

Consistent with the lack of an arrest, we observed that oncogene-expressing NAC-treated cells did not express senescence-associated beta-galactosidase (SA-beta-gal) activity, a common marker of cellular senescence establishment (Figure 1c). We previously proposed that oncogene-induced cell hyperproliferation ultimately leads to OIS as the consequence of DDR activation.^11^ We therefore tested whether ROS scavenging, by reducing cell proliferation, allows senescence avoidance by preventing the generation of DDR-activating DNA lesions. We observed that the accumulation in oncogenic Ras-expressing cells of both *γ*H2AX and 53BP1 foci were quantitatively reduced by NAC, but not NAA, treatment (Figure 1d).

We and others have previously proposed that oncogenes induce DDR activation by altering the DNA replication process, and no DDR is induced if oncogenic Ras is expressed in non-proliferating cells.^11, 12^ The involvement and potential contribution of ROS in these events is presently untested. We observed that expression of oncogenic Ras by the use of a lentiviral vector in proliferating or contact-inhibited quiescent cells leads to comparable robust increases in H-RasV12 expression and intracellular ROS levels (Supplementary Figures S1G–I). However, oncogenic Ras triggers DDR activation only in proliferating cells (Figure 1e; Supplementary Figures S1J and K). Thus, ROS become genotoxic only if they are allowed to exert their mitogenic function—that is, under these settings, if they are allowed to fuel hyperproliferation.

Overall, these results indicate that oncogene-induced ROS have mitogenic functions and their ability to generate DNA damage cannot be separated by their ability to promote cell hyperproliferation.

### Rac1 has an essential role in Ras-induced ROS production

We next attempted to elucidate the molecular pathway responsible for the generation of ROS within the pathway downstream of Ras activation. Rac1 has an important role in the transformation process. It is required for tumor development in K-RasV12-induced cancer murine models^27^ and it has been shown to regulate ROS levels.^28, 29^

**Figure 2:**
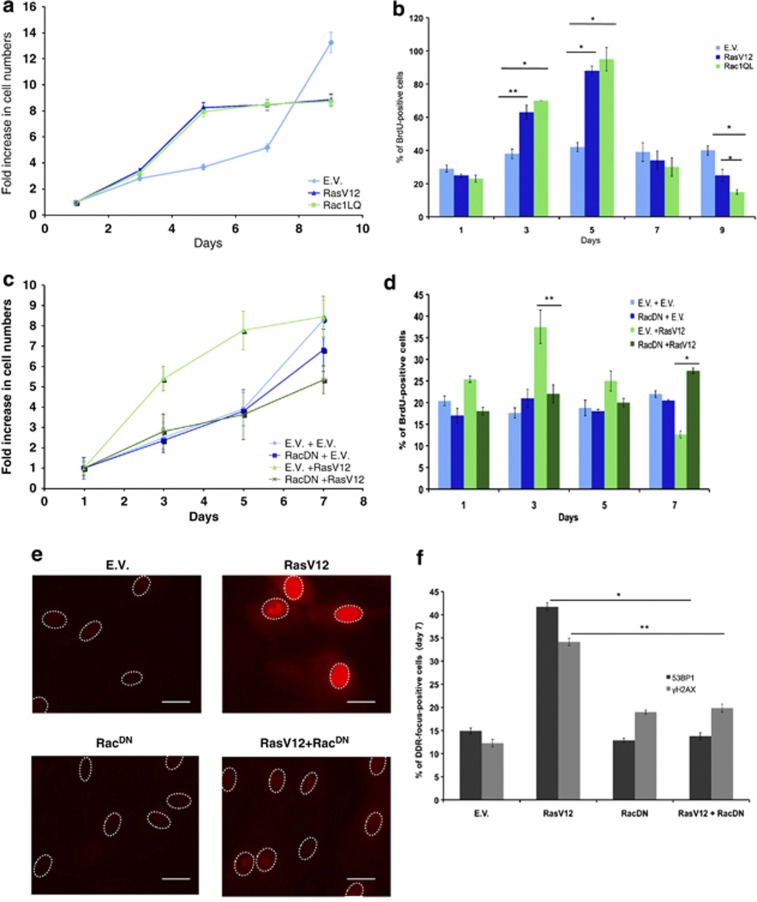
Rac mediates H-RasV12-induced ROS production and their effects. (**a**) NHFs were transduced with E.V. or H-RasV12- or activated Rac1 (Rac1QL)-expressing retroviruses. The growth curves show that activated Rac1QL induces hyperproliferation and cellular senescence in a manner very similar to RasV12. Proliferation is shown as the ratio of fold increase in cell numbers compared with day 1. (**b**) BrdU incorporation rates measured at the indicated time points of the growth curve shown in **a** demonstrate that Rac1QL expression mimics RasV12 expression. BrdU incorporation rates of RasV12- and Rac1-expressing cells are compared at days 3, 5 and 9 with respect to the E.V. Error bars indicate S.E.M. (*n*≥ 3), and differences are statistically significant (*P-value < 0.01, **P- value <0.05). (**c**) Expression of a dominant negative form of Rac1 (RacDN) prevents H-RasV12-induced hyperproliferation and OIS establishment. (**d**) BrdU incorporation rates measured at the indicated time points of the growth curve shown in **c** demonstrate that expression of RacDN prevents oncogene-induced increased DNA replication rates and replicative arrest associated with OIS. BrdU incorporation rates of RasV12-expressing cells are compared at days 3 and 7 with respect to RasV12-activated co-expressing RacDN cells. Error bars indicate S.E.M. (*n*≥ 3), and differences are statistically significant (*P-value < 0.01, **P -value<0.05). (**e**) RacDN expression in RasV12 cells reduces the level of oncogene-induced ROS as detected by DHE. Scale bar: 20 *μ*m. (**f**) Quantification of oncogene-induced DDR in the form of *γ*H2AX and 53BP1 nuclear foci of cells at the last time point of the growth curves shown in **c** indicates that DDR is reduced in RasV12-expressing cells upon co-expression of RacDN. The percentages of DDR in the form of *γ*H2AX and 53BP1 foci of cells are compared individually for RasV12-expressing cells between co-expression of E.V. and RacDN. Error bars indicate S.E.M. (*n*≥ 3), and differences are statistically significant (*P-value < 0.01 for 53BP1 foci; **P-value < 0.05 for *γ*H2AX foci). (See also Supplementary Figure S2)

We therefore transduced proliferating NHF with a constitutive active mutant form of Rac1 (Rac1QL) and tested whether its expression was sufficient to recapitulate the effects of oncogenic Ras described above. We observed that expression of Rac1QL leads to an increase in cellular ROS similar to H-RasV12 expression (Supplementary Figures S2A and B) and to a hyperproliferative growth phase, which is followed by DDR activation and growth arrest with kinetics and intensity that mirrored the observation following oncogenic Ras expression (Figures 2a and b). Rac1 was associated with genomic instability and cellular senescence previously,^30^ but it was never studied as a mediator within the context of hyperproliferative phase-modulating ROS levels.

In order to establish the involvement of Rac1 upon Ras activation, we expressed, together with H-RasV12, a mutant form of Rac1 (RacN17) previously reported to act as a dominant negative allele (RacDN) and to block the signals generated by Ras, including ROS production.^15^ We observed that co-expression of RacDN abolished the initial hyperproliferation induced by oncogenic Ras, as measured by cell numbers and the fraction of cells in the S phase, and allowed cells to further proliferate and incorporate BrdU (Figures 2c and d; Supplementary Figure S2E). In addition, ROS induction upon oncogenic Ras expression was markedly reduced upon co-expression of RacDN (Figure 2e; Supplementary Figure S2C). Consistent with our model of a causal link between ROS production, cell hyperproliferation and consequent DDR activation, the expression of RacDN also prevented DDR activation by oncogenic Ras (Figure 2f; Supplementary Figures S2D–H).

Thus, Rac1 is both necessary and sufficient to recapitulate the events that follow oncogenic Ras activation, namely ROS production, cell hyperproliferation, DDR activation and proliferative arrest.

### Nox4 has an essential role in Ras-induced ROS production

**Figure 3:**
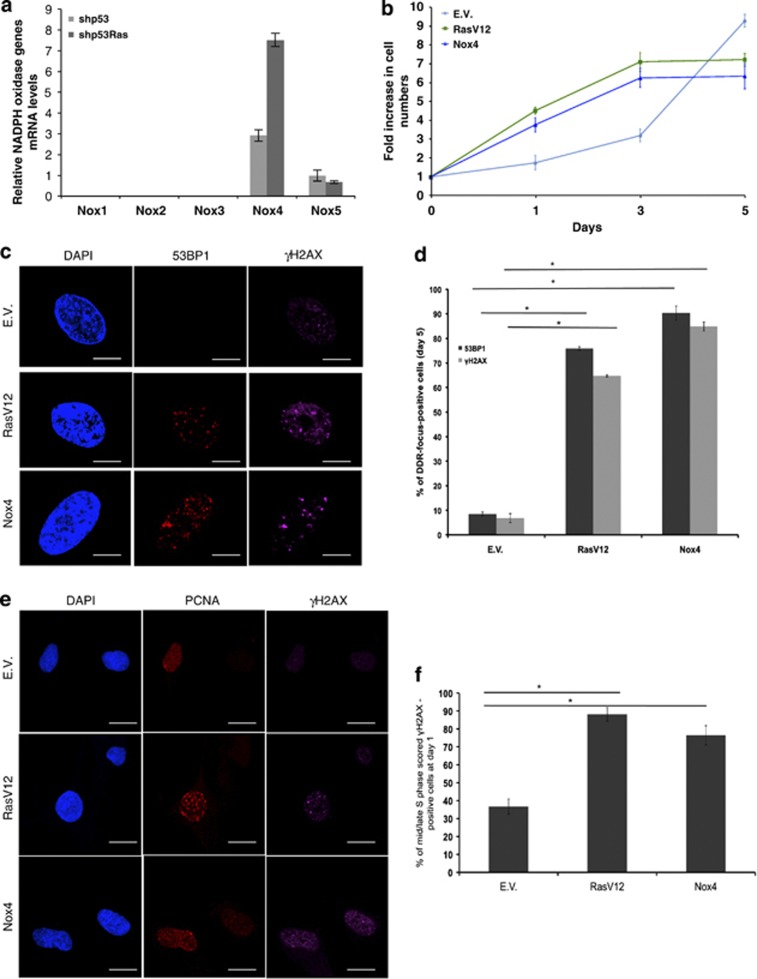
Nox4 is induced by H-RasV12 and its expression is sufficient to drive hyperproliferation, DDR activation and proliferative arrest. (**a**) qRT-PCR analysis of known human NOX gene paralogs shows that NOX4 is induced upon RasV12 expression. (**b**) Nox4 overexpression in NHFs is sufficient to induce hyperproliferation and a proliferative arrest similar to RasV12 expression. Proliferation is shown as the ratio of fold increase in cell numbers compared with day 0 (initial plating day) For further details, see Materials and Methods and also Supplementary Figure S3. (**c**) Nox4 overexpression triggers DDR to an extent similar to RasV12 expression. Confocal images of DDR markers immunostained for 53BP1 and *γ*H2AX in E.V., RasV12 and Nox4-transduced cells. The percentages of DDR in the form of *γ*H2AX and 53BP1 foci of cells are compared individually among RasV12- or Nox4-expressing cells with respect to E.V. Error bars indicate S.E.M. (*n*≥ 3), and differences are statistically significant (*P-value < 0.01) throughout the figure where stated. Scale bar: 2.8 *μ*m. (**d**) RasV12 or Nox4 expression induces similar levels of DDR as shown by quantification of DDR in the form of 53BP1 and *γ*H2AX foci in cells at the last time point of the growth curve in 3B. (**e**) Confocal images of DDR markers immunostained for *γ*H2AX in E.V., RasV12 and Nox4 lentiviral-transduced cells demonstrate that DDR activation was induced similarly in both RasV12 and Nox4 expressing cells. The mid or late S phase stage of the cell cycle was established by scoring nuclear PCNA staining patterns.^66^ Scale bar: 12 *μ*m. (**f**) RasV12 or Nox4 expression with respect to E.V. infected NHF cells induces similar levels of DNA replication stress as shown by the quantification of *γ*H2AX focus formation with cells having a mid or late S phase PCNA foci at day 1 of the growth curve in panel **b**. The percentages of DDR in the form of *γ*H2AX foci of cells are compared individually among RasV12- or Nox4-expressing cells with respect to E.V. Error bars indicate S.E.M. (*n*≥ 3), and differences are statistically significant (*P-value < 0.01) throughout the figure where stated

We next attempted to identify the enzyme responsible for ROS production upon oncogenic Ras expression and for the effects described above. NOXs are a family of enzymes, some members of which have been previously implicated in cell proliferation and cancer.^19, 31, 32^ As different cell types may express distinct sets of *NOX* genes, we first studied the expression levels of individual *NOX* genes (*NOX*1-5) upon oncogenic Ras expression. For this purpose we used NHF knocked down for p53, which does not senesce upon oncogene expression^11^ and thus grows at a constant rate and provides a cell system not affected by different proliferation rates. We observed that NOX4 was most robustly expressed in NHF among the *NOX* genes tested, and it was further induced upon oncogene activation (Figure 3a), consistent with previous reports.^33, 34^ The expression levels of individual NOXs are in line with^35^ levels observed in NHFs. NOX4 induction was further confirmed in wild-type NHF, both upon H-RasV12 and Rac1QL expression (Supplementary Figure S3A). Furthermore, we observed increased NOX activity as detected by a shift in the ratio of NADP+/NADPH levels in oncogenic Ras-expressing cells (Supplementary Figure S3B).

We thus probed the potential role of NOX4 in oncogenic Ras-induced hyperproliferation, ROS production, DDR activation and senescence establishment. First, we tested whether NOX4 overexpression could recapitulate the features of cells expressing oncogenic Ras. We observed that NOX4-overexpressing cells undergo an initial hyperproliferative phase, which culminates with a growth arrest that parallels the one observed upon oncogenic Ras expression (Figure 3b; Supplementary Figures S3B and C). In addition, both oncogenic Ras and NOX4 expression are associated with an increase in ROS levels (Supplementary Figures S3D and E) and induction of a robust DDR (Figures 3c and d). Importantly, both upon expression of oncogenic Ras and NOX4, DDR activation in the form of *γ*H2AX foci was induced in DNA replicating cells, as demonstrated by their staining for the DNA replication factor PCNA (Figures 3e and f), hence further highlighting the common DNA replication stress induced by these two genes.

Next, we tested the role of NOX4 as a ROS-producing enzyme downstream of oncogenic Ras. We observed that knockdown of NOX4 (Supplementary Figures S3 F and G) impairs cell proliferation and prevents oncogene-induced cell hyperproliferation. Lowered ROS levels in oncogene-expressing cells, reduced oncogene-induced DDR activation and prevention of SA-beta-gal activation (Supplementary Figures S3H–J) are consistent with the observed loss in the hyperproliferative phase. Reduced DDR activation is not due to a direct involvement of NOX4 in DDR signaling, as NOX4 overexpression or knockdown does not affect the extent of DDR activation upon exposure of cells to ionizing radiation (Supplementary Figures S3L–N).

Overall, these results are consistent with a model in which, upon oncogenic Ras expression, Nox4 is a key enzyme that produces ROS that fuel the observed oncogene-driven proliferative burst, which in turn causes DDR activation and ultimately establishment of cellular senescence.

### Pharmacological inhibition of Nox4 prevents the phenotypes associated with oncogenic Ras expression

Given the role of Nox4 in oncogenic Ras-induced proliferation, we tested the effects of specific pharmacological inhibitors on Nox4 enzymatic activity. We used two structurally distinct Nox4 small-molecule inhibitors (Nox4i1 and Nox4i2), previously identified and characterized in a cell-based assay for their ability to specifically inhibit ROS production by Nox4.^36^

**Figure 4:**
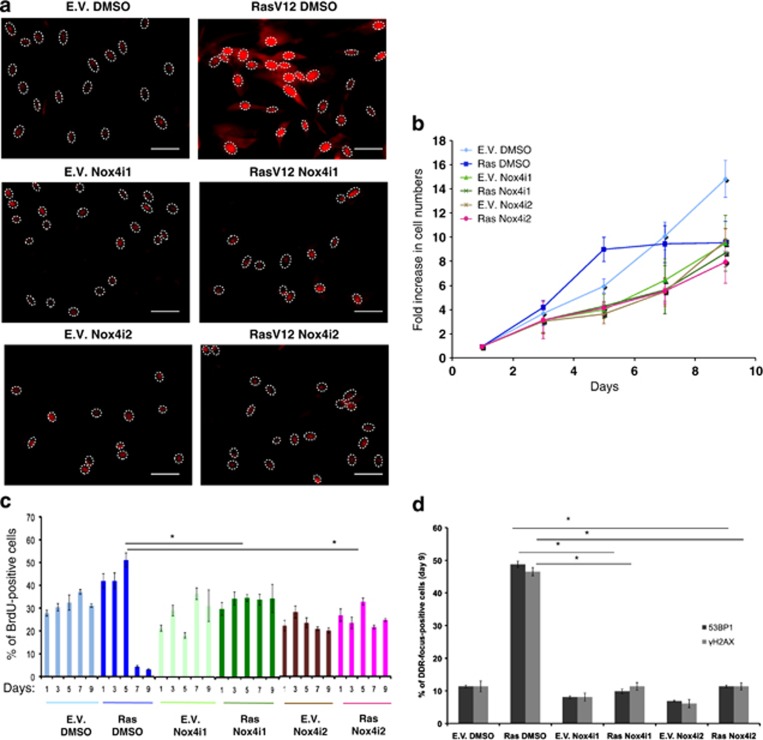
Pharmacological inhibition of Nox4 enzymatic activity prevents hyperproliferation by H-RasV12. (**a**) Nox4i treatment reduces ROS accumulation in RasV12 cells as detected by the DHE probe. Scale bar: 40 *μ*m. (See also Supplementary Figure S4A). (**b**) Two distinct NOX4 inhibitors (NOX4i1, NOX4i2), but not their vehicle (DMSO) prevent RasV12-induced hyperproliferation and OIS establishment. (**c**) BrdU incorporation rates measured at the indicated time points of the growth curve displayed in panel **a** show that both hyperproliferation and cellular senescence are abolished by Nox4i treatment. Differences in the rates of BrdU incorporation between RasV12-expressing cells treated by DMSO *versus* the ones treated by either Nox4i1 or Nox4i2 inhibitors at day 5 (the peak of the hyperproliferative phase) are statistically significant (*P-value < 0.01). (**d**) Immunostaining of cells at the last time point of the growth curves shown in **b** indicates that DDR activation is strongly reduced by Nox4i treatment. The percentages of DDR in the form of 53BP1 and *γ*H2AX foci of cells are compared individually among RasV12-expressing cells with respect to treatment using two different Nox4 inhibitors. Error bars indicate S.E.M. (*n*≥ 3), and differences are statistically significant (*P-value < 0.01) throughout the figure where stated

Nox4i were individually tested for their ability to prevent oncogenic Ras-induced ROS production, hyperproliferation and DDR activation. We observed that both Nox4 inhibitors were highly effective in suppressing ROS induction upon oncogenic Ras expression (Figure 4a; Supplementary Figure S4A). As superoxide anions in a cell quickly dismutate into the most stable hydrogen peroxide (H_2_O_2_) molecule, we used the hydrogen peroxide-specific peroxy orange 1 (PO1)^37^ probe to detect and measure these species. The use of this ROS probe confirmed the activities of Nox4i (Supplementary Figures S4B and C).

In addition, both inhibitors, used individually, suppressed oncogene-induced cell hyperproliferation, whereas empty vector-transduced cells were not significantly affected—this was observed both by measuring cell numbers and the fractions of BrdU-incorporating cells (Figures 4b and c). Consistent with our model, the prevention of cell hyperproliferation achieved by the use of Nox4i also prevented oncogene-induced DDR activation (Figure 4d; Supplementary Figures S4D and E).

These results show that the enzymatic inhibition of Nox4 prevents oncogenic Ras-induced ROS accumulation, cell hyperproliferation and DDR activation.

### ROS scavengers and Nox4 inhibition rescue the detrimental effects of systemic expression of oncogenic Ras on zebrafish

Oncogene-induced ROS have never been reported in a living animal, so far. To address this important issue, we exploited the transparency of *D. rerio* (zebrafish) larvae to attempt the detection of oncogene-induced ROS in a living vertebrate. We used a strain carrying a heat shock-inducible oncogenic form of human *H-RAS* fused to eGFP tg(hsp70I:eGFP-H-RASV12)io003. In this system, the induction of oncogenic Ras faithfully recapitulates some crucial features of Ras activation observed in other systems, including DDR activation and cellular senescence.^38^

**Figure 5:**
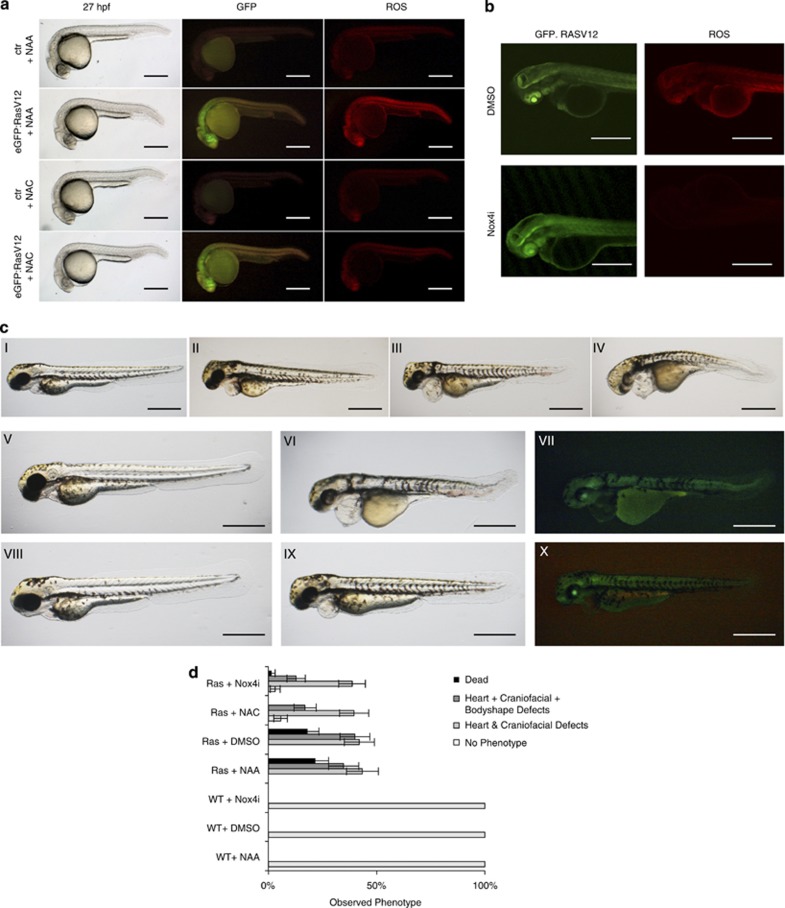
H-RasV12 induces ROS accumulation in living zebrafish larvae and ROS scavenging or NOX4 inhibition prevents Ras-induced larvae defects and death. (**a**) Ras-induced ROS can be detected in a living animal. A zebrafish strain carrying heat-inducible eGFP.H-RasV12 was heat-shocked and 27 h post-induction either control or eGFP.H-RASV12 transgenic animals were visualized by live imaging for GFP expression and ROS levels with the DHE probe. Scale bar: 100 *μ*m. (**b**) NOX4 inhibition reduces H-RasV12-induced ROS accumulation in zebrafish larvae as detected by live imaging by DHE. Scale bar: 200 *μ*m. (**c**) The observed phenotypes following heat shock (HS) in wild type (I) and H-RasV12 transgenics (II–IV) are displayed in each panel:I, normal animal; II, heart defects; III, heart and craniofacial defects; IV, heart, craniofacial and body defects; V, wild-type animal treated with DMSO after HS; VI, H-RasV12 transgenic animal treated with DMSO after HS; VII, the level of GFP-Ras in the larvae depicted in VI; VIII–X, heat-shocked larvae treated with 1–5 *μ*M of Nox4i; VIII, wild-type animal treated with Nox4i after HS; IX, H-RASV12 transgenic animal treated with Nox4i after HS; X, level of GFP-Ras in the larvae depicted in IX. Scale bar: 100 *μ*m. (**d**) The quantification of the occurrence of the phenotypes (demonstrated in **c**) following different treatments shows that ROS scavenging or Nox4 inhibition rescues the detrimental effects of RasV12 expression. Differences between the percentages of phenotypes observed in H-Rasv12 transgenics treated by NAA *versus* NAC and DMSO *versus* Nox4i are statistically significant scored for death and also heart, craniofacial and body defects (*P-value < 0.01)

On expression of oncogenic Ras in zebrafish larvae, we observed a robust induction and accumulation of ROS throughout the body of the animals (Figure 5a)—ROS were detected *in vivo* by adapting a protocol^39^ based on DHE. Treatment with NAC, but not NAA, prevented the increase in ROS production in heat-shocked H-Ras V12-expressing larvae (Figure 5a).

To probe whether the mechanisms leading to ROS production downstream of oncogenic Ras are conserved in fish, we investigated the involvement of Nox4 in this process. NOXs are still poorly characterized in zebrafish. *In silico* sequence homology searches identified three potential Nox4 orthologs in the zebrafish genome (Supplementary Figure S5A). To identify the effector of Ras in ROS production, we tested the mRNA levels of these three orthologs upon Ras activation *in vivo*. qRT-PCR analyses of mRNA transcripts showed that gene 1 was the most responsive, followed by gene 3, whereas expression of gene 2 did not change upon oncogenic Ras expression (Supplementary Figure S5B). Interestingly, the two zebrafish genes whose expression increased upon Ras activation, contain regions that are highly homologous to riboflavin synthase-like and NAD-binding domains, which have been directly involved in the enzymatic activity of human Nox4.^40^ These results suggest that Nox4 orthologs are conserved in zebrafish and H-RAS activation upregulates the expression of two of them.

Next, we tested whether the observed induction of Nox4 in zebrafish was relevant for oncogene-induced ROS production and whether inhibitors of human NOX4 were also effective in zebrafish in a whole living animal. We observed that treatment with Nox4i1 strongly reduced ROS levels in oncogene-expressing zebrafish larvae (Figure 5b), supporting the evolutionary conservation of the mechanisms of ROS production in zebrafish and the suitability of this novel *in vivo* model for studies of oncogene-induced ROS production and their effects.

On induction of H-RasV12 expression at 24 h post fertilization (h.p.f.), zebrafish larvae develop a range of phenotypes 3 days post fertilization (d.p.f.) that we classified into three groups according to the severity of the phenotype: dead; heart, craniofacial and body shape defects; and heart and craniofacial defects (Figure 5c (I–X)). To probe for the contribution of ROS to these defects, we tested the impact of ROS scavenging by NAC; NAA was used as a negative control. NAC strongly reduced the occurrence of death and all other phenotypes (Figure 5d), indicating that Ras-induced developmental defects are dependent on ROS production. Furthermore, we observed that the specific Nox4 inhibitor faithfully recapitulated the effect of the broad ROS scavenger NAC, indicating a crucial role of NOX4 in ROS production and in the induction of developmental defects and lethality.

Thus, oncogene-induced ROS can be detected in living vertebrates and their mechanism of action is evolutionary conserved from fish to humans. ROS accumulation *in vivo* causes death and developmental defects, which can be prevented by ROS inhibitors.

### NOX4 expression increases during the progression of K-Ras-driven pancreatic cancer

**Figure 6:**
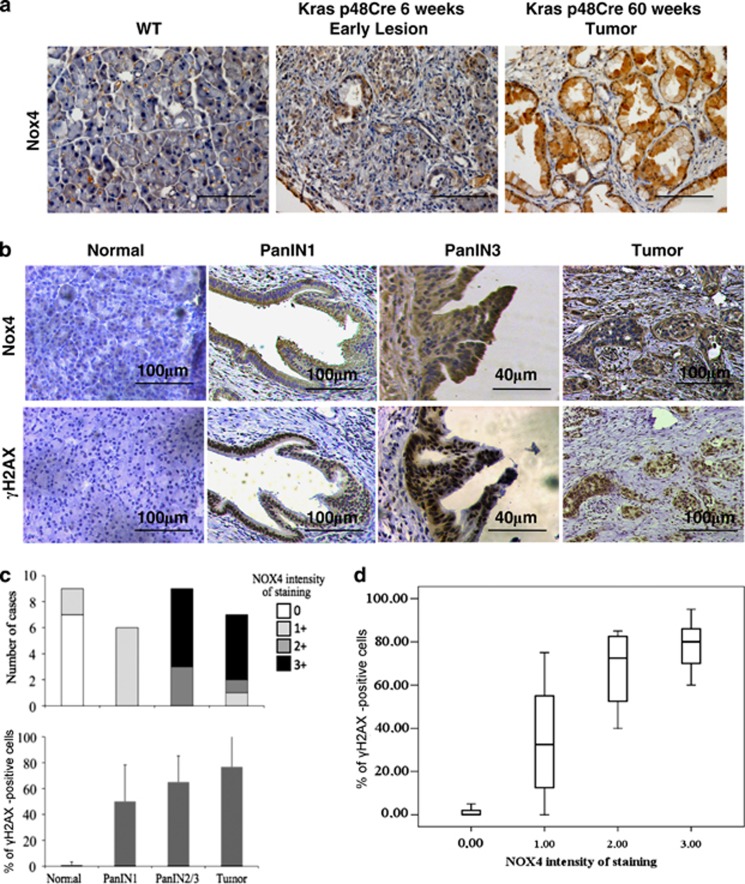
Increased NOX4 expression is associated with pancreatic cancer progression and correlates with DDR accumulation. (**a**) Immunohistochemical staining of NOX4 on sections of WT and p48-Cre; LSL-KrasG12D mouse pancreata. In normal WT pancreas, Nox4 is not detectable in acinal or ductal cells, the proposed cells of origin of PDAC. In p48-Cre, LSL-KrasG12D animals, NOX4 becomes detectably expressed already in early PanINs and more robustly in late invasive lesions. Scale bar: 60 *μ*m. (See also Supplementary Figure S6B). (**b**) NOX4 expression is undetectable by IHC in human normal pancreas, whereas its expression is upregulated in early panIN and late invasive tumors (upper panels). *γ*H2AX immunohistochemistry on consecutive sections shows a staining pattern overlapping with that of NOX4. Scale bars are indicated. (**c**) Quantification of the staining in **b**. Two independent pathologists scored the intensity of cytoplasmic NOX4 staining and the percentages of *γ*H2AX-positive nuclei in ducts, panINs and tumors. A scale from 0 (no staining) to 3+ (intense staining) was used. More than 50% of panIN3 and invasive lesions are strongly positive for NOX4. (**d**) NOX4 and *γ*H2AX stainings correlate across the full spectrum of pancreatic lesions, from panINs to invasive

Given the observed interplay between Nox4 and oncogenic Ras in cultured cells and in zebrafish, we investigated the role of Nox4 in human pancreatic cancer, an aggressive tumor type typically associated with activating Ras mutations.^41^ We first analyzed Nox4 expression in a well-established mouse model of pancreatic ductal adenocarcinoma (PDAC) (p48-Cre; LSL-KrasG12D) in which a conditional mutant K-Ras allele is activated by Cre recombinase in acinar and ductal cells leading to the progressive development of PDAC, thus faithfully recapitulating the progression of the human disease.^42^ We observed that while normal acini and ducts stained negative, or very weakly positive, for Nox4, increased expression was detectable in panINs (pancreatic intraepithelial neoplasia—the early and intermediate stages of pancreatic cancer) and PDAC lesions (Figure 6a)—Nox4 antibody specificity was tested in NHFs, murine and human sample sections (Supplementary Figures S6A–D; see also Materials and Methods). In addition, we studied Nox4 expression levels by qRT-PCR in the pancreas of a distinct but related model of pancreatic cancer driven by oncogenic Ras (Pdx1-Cre; LSL-KrasG12D)^42^ at different stages of tumorigenesis. We observed an increase in Nox4 mRNA levels (Supplementary Figure S6E) consistent with the IHC results (Figure 6a). These results indicate that oncogenic Ras activation in two genetically defined mouse models is associated with NOX4 induction, consistent with our observations in cultured cells.

We next studied ROS levels *in situ* in frozen tissue sections of pancreas of ‘Pdx1-Cre; LSL-KrasG12D' mice using the DHE probe, by a recently described approach.^43^ We observed that while normal tissue shows low ROS levels, oncogenic Ras activation leads to increased ROS levels (Supplementary Figures S6F and G). It has been proposed that K-Ras activation in pancreas promotes ROS detoxification through NRF2 and Nqo1, one of the targets of Nrf2.^44^ Indeed, we observed a mild induction of Nqo1 in these samples as detected by qRT-PCR and IHC (Supplementary Figures S6E, H and I), thus suggesting that Nqo1 is engaged. Thus oncogene activation leads to increased Nox4 expression and augmented ROS levels *in vivo*.

We next analyzed human PDAC samples for NOX4 expression. We observed a weak or absent signal in the normal tissue and a robust NOX4 upregulation in panINs (panIN1 and 3) and in invasive lesions, with signal intensities correlating with neoplastic stages (Figures 6b and c). Importantly, when consecutive sections were stained for *γ*H2AX, a very similar pattern was observed with *γ*H2AX and NOX4 signals strongly correlating (Figures 6c and d). These observations may not be restricted to pancreatic cancer, as analyses of data deposited in the Oncomine database indicated that Nox4 is upregulated in several cancer types when compared with their tissue of origin (Supplementary Figure S6J).^45^

Overall, these results point to NOX4 as an important mediator of oncogenic functions, specific for the neoplastic condition and common among several tumor types.

### Oncogenic Ras-expressing cells depend on ROS for proliferation

Our results show that in oncogene-expressing cells, ROS inhibition is an effective strategy to reduce hyperproliferation, prevent DDR activation and OIS. However, cancer cells have bypassed OIS by DDR inactivation and continue to show rampant proliferation together with high levels of ROS.^46, 47^ The role of ROS in settings, in which DDR is inactivated, and in fully transformed cells deserves attention.

First, we monitored ROS levels in DDR-deficient oncogene-expressing NHF either because knockdown for p53 (here referred to as BJ shp53 Ras^11^) or fully transformed NHF (BJELR; expressing oncogenic Ras), hTERT and SV40 early region;^48^ ROS were measured either by DHE (which preferentially measures superoxide anions, the direct product of Nox4) and also by CM-H_2_DCFDA, a commonly used ROS probe detecting mainly hydrogen peroxide but also hydroxyl radicals and peroxynitrite). By both approaches we observed increased levels of ROS upon oncogenic Ras expression, despite compromised DDR functions (Supplementary Figures S7A–D).

Next, we tested BJ shp53 Ras and BJELR and their respective control cell lines (BJ shp53 and BJhTERT) for their response to NAC, NAA, and the two NOX4 inhibitors. We observed that the decrease in ROS levels consistently and effectively reduced cell proliferation rates and BrdU incorporation specifically in cells expressing oncogenic Ras (Supplementary Figures S7 E–H). The negative impact of Nox4 inhibitors on proliferation was further substantiated by reduced levels of proliferative markers such as PCNA, CDC6 and MCM (Supplementary Figures S7I and L). Notably, the observed sensitivity of BJ shp53 Ras indicates that it is not the process of cell transformation *per se* that determines ROS dependency, rather it is the expression of the oncogene.

In a recent article,^49^ Nox4 was also involved in the induction of cytokines typically associated with the so-called senescence-associated secretory phenotype (SASP).^50^ Thus, we tested the impact of Nox4 inhibitors in shp53 Ras and BJELR cells on some key SASP components: IL1a, IL6 and IL8. We observed SASP was negatively regulated by these treatments, consistent with a causative role of Nox4 in SASP induction (Supplementary Figures S7J and M, consistent with ROS levels as shown in Supplementary Figures S7K and N).

**Figure 7:**
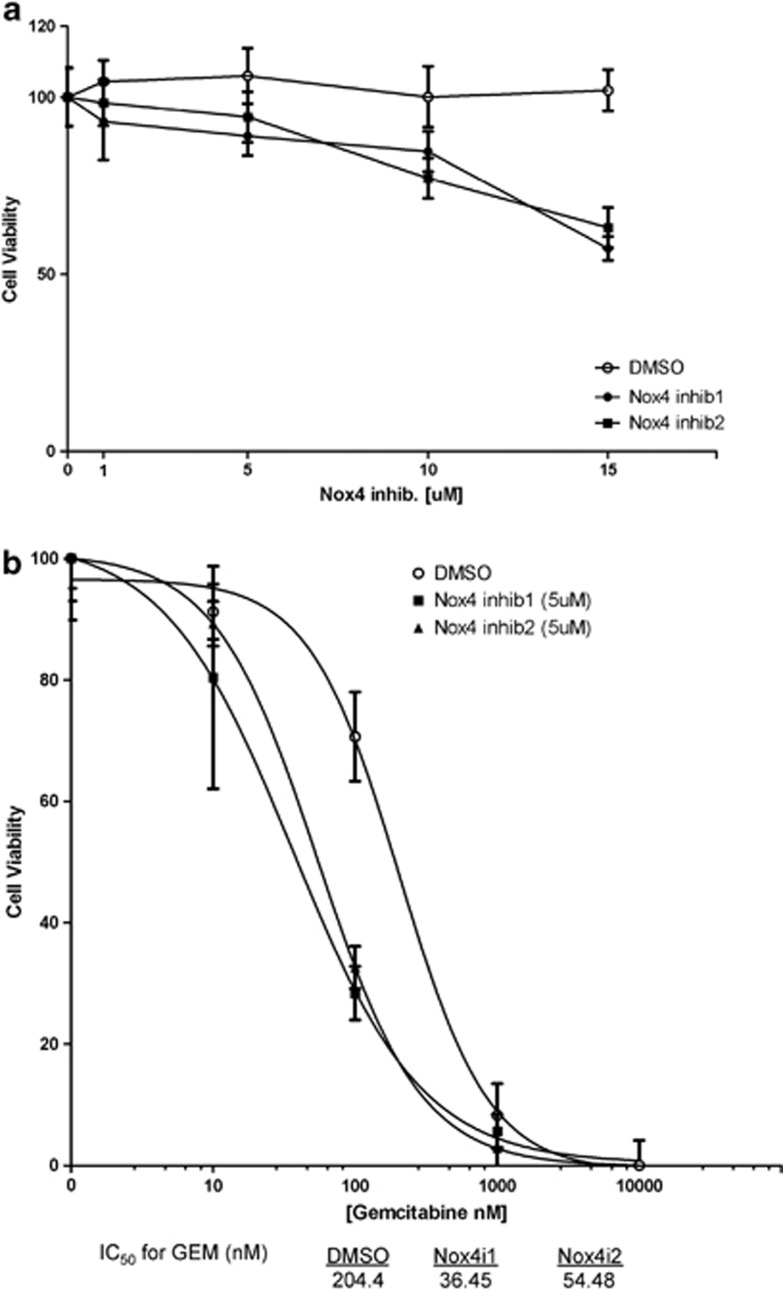
K-Ras mutant pancreatic cancer cell viability is dependent on Nox4. (**a**) Both Nox4i1 and Nox4i2, used individually, reduce cell viability of Panc1 cells. Panc1 cells were treated with an increasing dose of concentrations of Nox4is for 72 h. They were stained using the crystal violet assay and the viability of cells was quantitated. (**b**) Combination of gemcitabine and either Nox4i1 or Nox4i2 acts in a synergistic manner to reduce Panc1 cell viability. Panc1 cells were treated with increasing doses of GEM and different concentrations of Nox4is for 72 h. They were stained using the crystal violet assay and the viability of cells was quantitated. Here, we show the results of the assay at a 5-*μ*M concentration of Nox4is combined with a serial 10-fold dilution of GEM. IC50 values for GEM treatment together with DMSO, Nox4i1 and Nox4i2 are shown

Chemotherapy is still the standard of care for the treatment of several tumor types, and gemcitabine is a nucleoside analog widely adopted as a first-line treatment for pancreatic cancer.^51, 52^ Given the capacity of NOX4 inhibitors to reduce cell proliferation selectively in oncogene-expressing cells, we tested the impact of NOX inhibitors, alone and in combination with gemcitabine, on Panc1 cells—a human PDAC cell line carrying a mutant K-Ras allele.^53^ We observed that both Nox4 inhibitors, used individually, reduced cell viability of Panc1 cells as single agents, although moderately (Figures 7a). However, the combined use of either of them with gemcitabine displayed a strong synergistic impact on reduced cell viability and enhanced apoptosis and it increased the half-maximal inhibitory concentration (IC50) of gemcitabine by four- to sixfold (Figure 7b; Supplementary Figure S7O).

These results demonstrate that oncogene-induced ROS synthesis can be exploited by pharmacologically targeting Nox4 with specific inhibitors to achieve selective and potent cancer cell growth inhibition in synergy with standard care of therapeutic treatments.

## Discussion

Oncogene-induced ROS have been a complex matter in the literature and so far what has been established is limited to ROS scavenging reduces DDR and eventually commitment to cellular senescence. However, oncogene induced ROS modulating hyperproliferation, hence, engaging the DDR activation by fueling replicational stress has never been implicated or studied.

**Figure 8:**
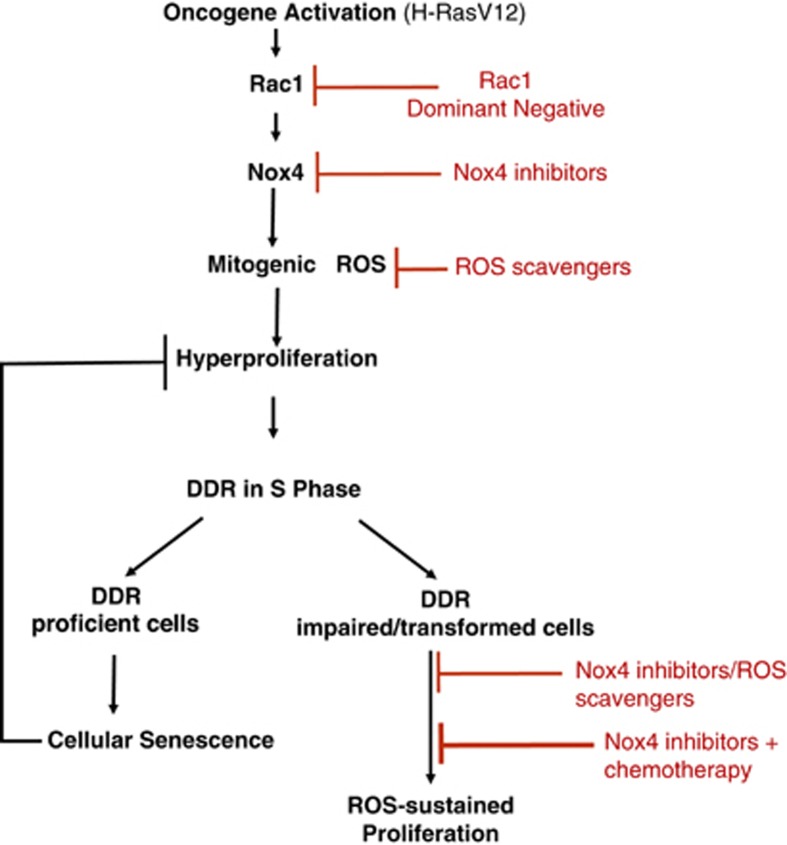
Scheme of origin and role of mitogenic ROS in cellular senescence and cancer

In summary, we have characterized a signaling cascade that, upon oncogenic RAS activation, engages RAC1 and NOX4, leading to the cellular accumulation of ROS that in turn have mitogenic functions and boost cell proliferation to the point of causing DDR activation and establishment of cellular senescence (Figure 8). A role of ROS in triggering DDR by mediating hyperproliferation was never suggested before. RAC1 and NOX4 are necessary mediators of these Ras effects, as shown by the demonstrated impact of a dominant negative allele of RAC1 and by NOX4 knockdown or chemical inhibition, which prevent ROS accumulation, cell hyperproliferation, DDR activation and cellular senescence. Furthermore, the individual expression of RAC1 and NOX4 demonstrated that these genes are also sufficient to recapitulate many of the events occurring following oncogenic RAS activation. The use of a transparent vertebrate (*D. rerio*) allowed us, for the first time to the best of our knowledge, to demonstrate oncogene-induced ROS accumulation in living animals, and thus to extend our observations beyond the use of cultured cells, providing a novel model system for the study of ROS *in vivo*. ROS induction in a complex tissue section such as a pancreatic tumor was further confirmed by DHE detection *in situ* in mice. It was shown in the same murine model that an antioxidant system mediated by Nrf2 and Nqo1 is activated upon oncogenic Ras activation.^44^ On the basis of our experimental results, it appears that this compensatory response to Ras-induced ROS production is not sufficient to fully buffer Ras-mediated ROS accumulation.

NOX4 levels are increased in a genetically defined mouse model of Ras-induced pancreatic cancer, as well as in human pancreatic tumors, which are known to exhibit aggressive growth. In these tumors, Nox4 expression correlates with DDR activation, and NOX4 and DDR activation both increase during cancer progression. Although Nox4 engagement and ROS accumulation had been previously linked to OIS and DDR in a different cell system,^34^ they have never been previously causally linked to oncogene-induced hyperproliferation and thus to a causative role in DDR activation through this mechanism.

Previously, we and others proposed that oncogene activation can be a DNA replication-dependent genotoxic event.^4, 11, 12, 54^ This is based on the observation that activation of an oncogene is followed by a burst of hyperproliferation that results in replicational stress, DDR activation and ultimately senescence. Several oncogenes (including Ras) are also known to induce ROS accumulation and experimental evidence indicates that ROS scavenging can prevent senescence establishment. Given the dual role of ROS as mitogenic signaling molecules and potential genotoxic agents, the instrumental role of ROS in senescence establishment has remained elusive.

Our results show that ROS induced by oncogenes are indeed signaling molecules with mitogenic functions. However, because of this very same property, by fueling hyperproliferation, they can be genotoxic and cause DNA damage generation and DDR activation. The observation that oncogene activation in non-proliferating cells does not cause DDR activation despite inducing high levels of ROS accumulation in the cells, indicate that the DDR-activating functions of ROS are dependent on ongoing DNA replication and their ability to boost it to the point of DNA damage generation.

Cell transformation is associated with OIS bypass and ongoing rampant proliferation. We observed that oncogene-expressing cells that have bypassed senescence by DDR inactivation and become transformed are particularly sensitive to ROS scavenging or specific NOX4 inhibition. This is likely owing to the fact that the survival of these cells depends on the mitogenic drive of the oncogene to contrast the residual DDR activities and DNA damage accumulation of cancer cells. Thus, any impairment in the synthesis or accumulation of mitogenic ROS, the ‘fuel of proliferation', may cause a reduction in proliferative ability in treated cells. Oncogenes have been shown to be not only necessary for cancer development, but also for its maintenance.^55^ Our results suggest that oncogene dependency in fact translates into ROS (and hence NOX4) dependency. The additional observation of a strongly synergic effect of NOX4 inhibition and DNA damage generation by gemcitabine on a pancreatic cancer cell line points to a potential translational value of our findings that deserves further exploration (Figure 8).

The observation of low levels of NOX4 expression in normal tissue *versus* precancerous and cancerous lesions further increases its appeal as a therapeutic target. A recurrent downside of targeting growth and survival pathways is the achievement of an efficacy window in order to eliminate only tumor cells, but not their normal neighbors. Although ROS is a global modulator of proliferation, ROS also have a critical role in regeneration as demonstrated elegantly in the amphibian tail regrowth.^56^ However, excessive ROS inhibits regeneration and promote carcinogenesis.^57^ We achieved a reduction in proliferation of oncogenic Ras cells while minimally, if at all, perturbing the normal cell compartment; this was particularly evident in the zebrafish model. Importantly, this observation supports the notion that Nox4 inhibitors may not affect normal tissue homeostasis, while exerting their effects on oncogenic Ras-expressing cells without disturbing the balance.

Given the differential expression of Nox4 in several cancer cell types, therapies targeting Nox4 specifically show promise when combined with existing antimetabolites or cytotoxic drugs. In addition to our results, a recent study shows that this can be extrapolated to renal cancer carcinoma,^58^ where Nox4 contributes chemoresistance by modulating antiapoptotic signaling and silencing Nox4 significantly lowers the IC50 values for chemotherapic drugs. Additional supporting evidence of selective pressure on DDR genes comes from a recent large-scale study. Using a combination of next-generation exome sequencing and copy number variant analysis from patients with PDAC, the authors reveal that ATM is often mutated.^59^

Finally, it is fair to remark that the practice of ROS scavenging and antioxidant treatments has a chequered history in terms of clinical benefits in cancer therapy.^60, 61^ We believe that our results may provide an additional perspective and contribute a fresh interpretation. Although we showed that ROS scavenging or inhibition reduces the proliferation of cancer cells, it is important to bear in mind that we also observed that ROS scavenging or Nox4 inhibition, in oncogene-expressing cells that have not yet accumulated DNA damage or bypassed OIS, may in fact allow these oncogene-expressing cells to proliferate without ‘hyperproliferating' (see Figure 1a). This may allow treated cells to expand without incurring in OIS establishment caused by DNA damage generation. Under these conditions, these treatments cannot be considered beneficial, as they ultimately cause the expansion of cells bearing an activated oncogene. Thus, according to the stage of a neoplastic lesion, ROS scavenging may allow oncogene-expressing cells to multiply or reduce in number. Evidence deriving from independent studies has concluded that oncogene-driven proliferation that occurs ‘below the radar' may allow the expansion of oncogene-expressing cells without engaging the DDR.^62^ Thus, cancer stage, its genetic make up, DNA damage accumulation and DDR proficiency, are parameters that may contribute to compound the clinical response to therapies based on ROS manipulations.

## Materials and Methods

### Cell culture

BJ (ATCC, Manassas, VA, USA), BJ hTERT (Clontech or they are produced by retroviral transduction of hTERT human normal fibroblast (NHF)) cells were grown under standard tissue-culture conditions (37 °C, 5% CO2) in MEM Glutamax 1X (Gibco-Invitrogen, Monza, Italy) supplemented with 10% fetal bovine serum, 1% non-essential aminoacids, 1% Na pyruvate, 1% penicillin/streptomycin. BJELR^48^ (a kind gift from William C Hahn, Dana-Farber Cancer Institute, Boston, MA, USA) were grown under standard tissue-culture conditions (37 °C, 5% CO2) in DMEM: M199 (4 : 1) supplemented with 10% fetal bovine serum, 1% Na pyruvate, 1% penicillin/streptomycin, 25 mM Hepes pH 7.5, 1% L-glutamine. For growth curves, cells were plated in triplicates at 5 × 10^4^ per well in six-well plates and were counted every other day.

### Cell treatments

NAC andNAA (Sigma-Aldrich, St. Louis, MO, USA) were freshly added every 48 h to the culture medium at 5 mM final concentration. H2O2 was used used at 1 mM concentration for 1 h. Nox4 inhibitors, VCC300991 : 01 and VCC444973 : 02,^36^ were synthesized by Vichem Chemie Ltd, Budapest, Hungary and were freshly added every 48 h at 5 *μ*M final concentrations (unless otherwise indicated). Gemcitabine was purchased from Sigma-Aldrich (Gemcitabine hydrochloride, G6423). All drugs were diluted in complete growth medium (DMEM supplemented with supplemented with 10% fetal bovine serum and 1% L-Glutamine).

### Plasmids

pBABE-Puro H-RasV12, pBABE-Hygro H-RasV12, pRETROSUPER shp53, Lenti GFP and Lenti H-RasV12 and corresponding empty vectors were used as described in.^11^ pLKO.1 shNox4 and pLKO.1-puro were obtained from the RNAi Consortium (Broad Institute, a kind gift from William C Hahn, Dana-Farber Cancer Institute). Lenti Nox4 was a kind gift from Ulla Knaus (UCD Conway Institute of Biomolecular and Biomedical Research, Dublin, Ireland).^63^ pBABE Rac1QL and pBABE RacN17 were kind gifts from Giorgio Scita (IFOM Foundation, Milan, Italy).

### Retroviral and lentiviral transduction

Amphotrophic phoenix viral packaging cells were transfected with the vector of interest for retroviral particle production by the calcium phosphate precipitation method. After transfection, supernatants containing viral particles were collected and used to transduce target cells as in Di Micco *et al.*^11^ Lentiviral particles were produced by transfecting HEK293T cells using the calcium phosphate method with pMDL, pVSVG, pRVS-REV plasmids and the vector of interest.

### Proliferation assay

BJ hTERT cells were seeded at a density of 5 × 10^4^ in triplicates on six-well plates the day after the infection. Cell proliferation over time was measured by counting the number of cells at indicated time points. Relative growth rate is plotted by assigning 1 to the first day of the counting and calculating the relative fold increase. When lentiviruses were used instead of retroviral particles, the effects of the hyperproliferative phase is observed since cells plating at day 0. Therefore, we assigned 1 to this day and calculated the fold increase in cell numbers as described above. In Supplementary Figure S1B, cells were selected for drug resistance and only then plated for proliferation analyses.

### BrdU incorporation assay

Cells plated on coverslips were incubated with BrdU (SIGMA, St. Louis, MO, USA) at 10 *μ*g/ml concentration for 6 h. Cells were fixed with 4% paraformaldehyde for 10 min followed by a permeabilization with 0.2% Triton X-100 for 10 min at room temperature. After blocking, cells were incubated with a mixture of BrdU antibody and DNase in DNase buffer containing MgCl_2_ for 45 min at room temperature. Coverslips were washed and incubated with a secondary antibody and then stained by DAPI before being mounted with Mowiol. At least 100–300 cells were analyzed for time point.

### Immunofluorescence microscopy

Cells were fixed with 1 : 1 methanol/acetone solution for 2 min at room temperature for PCNA, Nox4 and all other DDR markers staining. PCNA and *γ*H2AX costaining was preceded by *in situ* cell fractionation with pre-extraction.^64^ In case of immunofluorescence against Ras, cells were fixed with 4% paraformaldehyde for 10 min and permeabilized with 0.2% Triton X-100 for 10 min at room temperatures. Images were acquired by a wide-field Olympus Biosystems Microscope BX71 (Olympus Italia Srl, Milano, Italy) and analyzed by ImageJ software. Confocal sections were obtained with a Leica TCS SP2 AOBS confocal laser microscope (Leica Microsystems S.r.l., Milano, Italy) by sequential scanning. Comparative immunofluorescence analyses were performed in parallel with identical acquisition parameters and analysis; 100–300 cells were screened for each antigen. A threshold value was used to distinguish inidividual foci for each set of experiment using Image J software <http://rsbweb.nih.gov/ij/>. Histograms shown represent the percentage of cells showing ≥2 foci for each antigen analyzed.

### ROS detection

Antimycin A (Sigma-Aldrich), an inhibitor of respiration complex III, was used as a positive control to induce ROS production. Cells on coverslips were incubated with antimycin A at 10 *μ*g/ml for 30 min at 37 °C before adding the ROS probe DHE. All experiments included a sample incubated without the probe, as a negative control to check for autofluorescence. Cells were treated with DHE (Molecular Probes, Invitrogen, Monza, Italy) at a final concentration 5 *μ*M for 30 min at 37 °C, and washed with PBS before live imaging either by confocal or wide-field microscopes without fixation. ‘FITC excitation-CY3 emission' was performed by using a bandpass filter 470–495 nm (FITC excitation), a dichroic mirror (505 nm) and a highpass filter (520 nm). These filters allow specific detection of ROS also in GFP-expressing cells and the whole animals. All images were obtained within 30 min post treatment. Peroxy orange 1 (PO1) is a synthetic chemical probe, which is engineered for fluorescence detection of H_2_O_2_ in living cells upon oxidative stress. Cells were treated with PO1 at a final concentration of 5 *μ*M for 30 min at 37 °C, and washed with PBS before live imaging either by confocal or wide-field microscopes without fixation.

In zebrafish experiments, DHE was dissolved in DMSO and used at a final concentration of 10 *μ*M in fish water in the presence of 0.1% DMSO. Zebrafish were incubated with the probe for 15 min in a dark chamber, rinsed in fish water three times and then immediately imaged under a Nikon (Milan, Italy) stereomicroscope equipped with epifluorescence and filters for GFP and DS-Red. As controls, we used non-transgenic siblings that were heat-shocked alongside.

In mouse pancreatic sections, DHE was used at a final concentration of 30 *μ*M on tissues embedded in OCT (Optimum Cutting Temperature) cryomolds, which were cut in 8-*μ*m sections. Sections were incubated for 7 min at 37 °C in dark and washed twice carefully by PBS. Images were taken immediately after mounting them with vectashield containing DAPI. Fluorescence intensity of at least 100 nuclei per sample was scored in at least two mice per each experimental condition.^43^ As controls, we used K-Ras LSL no cre murine sections.

All the quantifications were performed using Image J software <http://rsbweb.nih.gov/ij/>. Each image was background subtracted and fluorescence mean intensity was measured. A mask has been used to create a region of interest (ROI) around each cell, where the mean intensity value was calculated. Nuclei and the GFP diffused signal were used as masks, in case of DHE and PO1 probes, respectively. Fluorescence intensity of at least 60–100 cells per sample in total was scored in at least three independent coverslips per each experimental condition. The average mean intensity was normalized for the control condition and at least three different replicates were used for each condition. Error bars represent±S.D.

ROS detection using CM-H_2_DCFDA was performed according to the protocol^65^ using a 96-well plate reader. 1 × 10^4^ cells per well were plated a day before the experiment. Drug treatments were carried out as in the rest of the experiments (that is, final concentrations 5 mM NAA, NAC and 5 *μ*M DMSO or Nox4i) for 1 h. Fluorescence was detected by a plate reader using excitation at 485 nm and emission at 525 nm wavelengths. Each experiment includes controls such as unloaded (no CM-H_2_DCFDA) and untreated (no drug) or unloaded and treated cells. Medium without phenol red was used to minimize interference with CM-H_2_DCFDA. Average mean value was calculated by using 10 replicates for each condition. Error bars represent±S.D.

### NADP/NADPH assay

NADP+ and NADPH levels were measured from cells using a NADP/NADPH Assay Kit (Abcam, Cambridge, UK; ab65349) as in Myant *et al.*^43^ A standard time course curve was performed at 10 minutes intervals to determine optimal incubation time (30 min at room temperature) for analysis. NADP and NADPH levels in total lysate were calculated by comparison with the standard curve. Protein concentration was determined for each samples and values represented as pmol NADP/NADPH per *μ*g filtered lysate.

### Quantitative real-time PCR

Total RNA was isolated from cells using RNAeasy (Qiagen Srl., Milan, Italy) according to the manufacturer's instructions and treated with RNase-free DNAse (Qiagen Srl.) before reverse transcription. In zebrafish and mouse sections, total RNA was isolated using Trizol (Invitrogen) followed by RNAeasy (Qiagen Srl.) kit. For each condition to extract RNA six fish and two slices of 10-*μ*m sections were used in zebrafish and mouse experiments, respectively. cDNA was generated using the Superscript VILO Reverse Transcriptase (Invitrogen). The cDNA was used as a template in real-time quantitative PCR reactions with specific primers on a Roche LightCycler 480 Sequence Detection System (Roche Applied Science, Monza, Italy). The reactions were prepared using SyBR Green reaction mix from Roche. Ribosomal protein P0 (*RPP0*) was used as a control gene for normalization. Primer sequences for qRT-PCR:

RPPO-fwd, 5′-TTCATTGTGGGAGCAGAC-3′ RPPO-rev, 5′-CAGCAGTTTCTCCAGAGC-3′ Nox1-fwd, 5′-AAGGATCCTCCGGTTTTACC-3′ Nox1-rev, 5′-TTTGGATGGGTGCATAACAA-3′ Nox2-fwd, 5′-GGTTTTGGCGATCTCAACAG- 3′ Nox2-rev, 5′-CGATGGTTTTGAAAGGGTGA-3′ Nox3-fwd, 5′-AGTTCAAGCAGATTGCCTACAA-3′ Nox3-rev, 5′-CGAGAGAGCTTTAGGTCCACA-3′ Nox4-fwd, 5′-GCTGACGTTGCATGTTTCAG-3′ Nox4-rev, 5′-CGGGAGGGTGGGTATCTAA-3′ Nox5-fwd, 5′- CGTCTGTGCCGGCTTATC-3′ Nox5-rev, 5′-CCAATTCCAGATACAACATGACTG-3′ PCNA-fwd, 5′-TGGAGAACTTGGAAATGGAAA-3′ PCNA-rev, 5′-GAACTGGTTCATTCATCTCTATG-3′ CDC6-fwd, 5′-CCTGTTCTCCTCGTGTAAAAGC-3′ CDC6-rev, 5′-GTGTTGCATAGGTTGTCATCG-3′ MCM6-fwd, 5′-acagctaagagccaatttctcaa-3′ MCM6-rev, 5′-ggacgctttaccactggtgt-3′ IL1A-fwd, 5′-GGTTGAGTTTAAGCCAATCCA-5′ IL1A-rev, 5′-TGCTGACCTAGGCTTGATGA-3′ IL6-fwd, 5′-GATGAGTACAAAAGTCCTGATCCA-3′ IL6-rev, 5′-CTGCAGCCACTGGTTCTGT-3′ IL8-fwd, 5′-GAGCACTCCATAAGGCACAAA-3′ IL8-rev, 5′-ATGGTTCCTTCCGGTGGT-3′ ZebraFish Nox4 Gene1-fwd, 5′-GCTTTACACATTGGGAGGAATC-3′ ZebraFish Nox4 Gene1-rev, 5′-CCGTTCCGTCATCCAAGT-3′ ZebraFish Nox4 Gene2-fwd, 5′-GCGATCTCGGACTGATTGA-3′ ZebraFish Nox4 Gene2-rev, 5′-CCCGTTAGCGTGGTTTGT-3′ ZebraFish Nox4 Gene3-fwd, 5′-TTGTCGGCTTCACATCCAT-3′ ZebraFish Nox4 Gene3-rev, 5′-GTCCCAAGCGTCTCTCACA-3′. Mouse Nox4-fwd, 5′-ttgtgaagatttgcctggaa-3′ Mouse Nox4-rev, 5′-aaggcacaaaggtccagaaa-3′ Mouse Nqo1-fwd, 5′-agcgttcggtattacgatcc-3′ Mouse Nqo1-rev, 5′-agtacaatcagggctcttctcg-3′.

All the primers were synthesized by Sigma-Aldrich.

### Antibodies

Anti-*γ*H2AX (Upstate Biotechnology, Millipore, Billerica, MA, USA; 1 : 200 for immunofluorescence and IHC); anti-53BP1(1 : 200 for immunofluorescence; Upstate Biotechnology); anti-BrdU (Becton Dickinson, BD Italia, Milan, Italy; 1 : 20 for immunofluorescence); anti-*γ*H2AX (Abcam;1 : 200 for immunofluorescence for PCNA experiments); anti-phosphoATM (Rockland, Gilbertsville, PA, USA; 1 : 200 for immunofluroescence); anti-phosphoS/TQ (Cell Signalling Technology, Danvers, MA, USA; 1 : 100 for immunofluorescence); anti-PCNA (Santa Cruz, Heidelberg, Germany; 1 : 200 for immunofluorescence); anti-Ras (BD Transduction Laboratories, BD Europe, Buccinasco, Italy; 1 : 200 for immunofluorescence); anti-NOX4 (Novus Biologicals NB110-58851, Novus Europe, Cambridge, UK; 1 : 200 for immunohistochemistry and immunofluorescence); anti-cleaved caspase 3 (Cell Signaling Technology, 1 : 500 for western blotting); anti-vinculin (Sigma-Aldrich, 1 : 10000). Samples from normal renal parenchyma were used as a positive control for NOX4 staining in mice experiments. A non-immune immunoglobulin of the same isotype was used as a negative control.

### Immunohistochemistry

Mouse samples: IHC analysis was performed on formalin-fixed paraffin-embedded samples from Kras-LSL p48-Cre mice as above except the antigen retrieval step, which was carried out using 0.25 mM EDTA pH 8.0 for four cycles of 5–6 min at 95 °C. Both Nox4 and secondary antibodies used were tested on tissues and cells for specificity (Supplementary Figures S6B and C).

Human samples: IHC analysis was performed on formalin-fixed paraffin-embedded samples from pancreatoduodenectomies. Briefly, paraffin sections, 5-mm thick, were mounted on poly-L-lysine-coated slides, dewaxed, rehydrated and incubated for 15 min with 3% hydrogen peroxide to quench the endogenous peroxidase activity. Antigen retrieval was carried out using 10 mM citric acid buffer pH 6.0 in a steamer for 15 min. The second-generation polymer method (UltraVision LP, LabVision, Thermofischer, Waltham, MA, USA) was used for signal detection according to the manufacturer's instructions. For color development, we used 3,30-diaminobenzidine tetrahydrochloride (DAB) (LabVision) and hematoxylin as counterstain. The samples are archival material and all the studies were conducted under the supervision and approval of the Local Ethical Committee of the Medical School of the University of Athens, following the requirements of the Helsinki Declaration as revised in 1983.

All the stainings were evaluated by two independent pathologists. The intensity of cytoplasmic NOX4 staining was evaluated in a semi-quantitative manner with a range from 0 to 3+. *γ*H2AX staining was evaluated quantitatively as the percentage of positively stained nuclei. An average of 500 cells were evaluated at × 400 magnification in each case. Mean *γ*H2AX expression and S.D. in all cases are shown in Figure 6d.

### Transgenic fish

The tg(hsp70I:eGFP-H-RASV12)io003 zebrafish transgenic line carrying a heat shock-inducible oncogenic form of human H-RAS fused to eGFP^38^ was used in all experiments. The induction of oncogenic Ras was obtained with a 20 min incubation at 39 °C at 24 h.p.f. Three hours later, the induction of Ras expression was confirmed under a fluorescent stereomicroscope. Induced zebrafish larvae continue to express eGFP-H-RASV12 for at least 5 days.

Phenotypic classification of zebrafish: the phenotype of treated larvae was assessed at 72 h.p.f. We classified the treated larvae as: unaffected (no phenotype), mild (heart and craniofacial defects), severe (heart, craniofacial and body shape defects) and dead.

All experiments with mice and zebrafish were performed in accordance with the guidelines established in the Principles of Laboratory Animal Care (directive 86/609/EEC) and approved by the Italian Ministry of Health.

### Drug combination treatment

2 × 10^3^ Panc1 cells per well were seeded into 96-well plates, allowed to grow for 24 h, and treated with drug combinations for 72 h. At the end of the assay, surviving cells were stained by crystal violet, which was then dissolved in 30% acetic acid and quantified by absorbance at 540 nm using a 96-well plate reader. Drug concentration was plotted *versus* normalized response. The least-squares curve fit was chosen to compute IC50s and inhibition curves. All analyses were performed in Prism (Graphpad Software, La Jolla, CA, USA).

### Statistical analysis

Results are shown as means±S.D. or S.E.M., as indicated. *P*-values were calculated by Student's two-tailed *t*-test. SPSS Statistics v17.0 software (Chicago, IL, USA) was used for the statistical analysis of the immunohistochemical data.

## Abbreviations

ROS: Reactive oxygen species
OIS: Oncogene-induced senescence
DDR: DNA damage response
Nox: NADPH oxidase
NHF: Normal human fibroblast
NAC: *N*-acetyl-cysteine
NAA: *N*-acetyl-alanine
DHE: Dihydroethidium
PO1: Peroxy orange 1
Nox4i: NADPH oxidase 4 inhibitor
BrdU: Bromodeoxyuridine
qRT-PCR: Quantitative real-time PCR
PanIN: Pancreatic intraepithelial neoplasia
PDAC: Pancreatic ductal adenocarcinoma
Pdx1: Pancreatic and duodenal homeobox 1
GEM: Gemcitabine
