# Supplementary material for: Oncogene-induced reactive oxygen species fuel hyperproliferation and DNA damage response activation
Source: Cell Death Differ. 2014 Feb 28;21(6):998–1012. doi: 10.1038/cdd.2014.16 (PMC4013514; doi:10.1038/cdd.2014.16)

Supplementary Figure Legends

Supplementary Figure 1

H-RasV12 expression increases ROS production and ROS scavenging prevents DDR in H-RasV12 cells.

1A NHFs were transduced with either empty vector (E.V.) or H-RasV12-expressing (Ras) retroviruses. Cells were treated with a broad-specificity ROS scavenger NAC (N- acetyl-L-cysteine) or a related inactive compound NAA (N-Acetyl-L-alanine). ROS levels were detected using DHE as a probe by live cell imaging. NAC treatment reduces ROS signal, whereas NAA treatment does not. Antimycin A, an inhibitor of respiration complex III, was used on untransfected NHF as a positive control for robust ROS production. Scale bar: 40μm.

1B Quantification of DHE signal as in panel 1A. Differences are statistically significant (*P value < 0.01).

1C ROS mediate H-RasV12-induced cellular senescence. NHFs were transduced with either empty vector (E.V.) or H-RasV12-expressing retroviruses. Cells were first selected for retrovirus-encoded drug resistance and then monitored for 17 days. ROS scavenging by NAC (added freshly to the media every 48 hours), but not NAA, prevents oncogene- induced cellular senescence establishment and allows cell proliferation, as measured by cell numbers. Hyperproliferative phase cannot be appreciated because here cells were first selected for puromycin resistance and thus growth curve was started after hyperproliferation occurred – see scheme in S1E.

1D Quantification of BrdU incorporation rates of the cells of the last time point of the growth curve shown in 1C confirms that NAC prevents OIS establishment in H-RasV12- expressing cells.

1E Schematic representation of experimental design for the growth curve shown in S1C.

1F Schematic representation of experimental design for the growth curve shown in Figure 1A. In order to best appreciate the hyperproliferative phase, cells were plated right after retroviral infection and without selection. All the growth curves shown in this manuscript (apart from S1C) were performed in this way.

1G Immunostaining of Ras in H-RasV12 in proliferating or quiescent NHFs shows comparable levels of Ras expression. Scale bar: 40μm.

1H H-RasV12 expression leads to similar rates of ROS accumulation, as detected by DHE, in proliferating or contact-inhibited quiescent cells. Scale bar: 40μm.

1I Quantification of DHE signal in panel S1H using Image J confirms similar levels of ROS accumulation in proliferating or contact-inhibited quiescent cells. The difference between proliferating or contact-inhibited quiescent cells is not statistically significant (n.s.) (p value > 0.05).

1J H-RasV12-induced ROS do not generate DNA damage in the absence of DNA replication. Quantification of immunostaining of DDR markers in the form of 53BP1 and γH2AX foci in proliferating (Pro) or quiescent (Q) E.V. or H-RasV12 transduced cells. Differences are statistically significant (**P* value < 0.01).

1K BrdU incorporation rates of proliferating or contact-inhibited quiescent NHFs. (**P* value < 0.01).

Supplementary Figure 2

Activated Rac1 (Rac1QL) induces ROS production and DDR activation to an extent similar to H-RasV12-expressing cells.

2A ROS production as detected by live imaging with DHE probe in E.V.-, H-RasV12- and Rac1QL-transduced cells. Scale bar: 20μm.

2B Quantification of DHE signal as in panel S2A. Differences are statistically significant (**P* value < 0.01).

2C Quantification of DHE signal as in Figure 2E. Differences are statistically significant (**P* value < 0.01).

2D Quantification of immunostaining of DDR foci markers in the form of pATM, pS/TQ, 53BP1 and γH2AX in E.V.-, H-RasV12- and Rac1QL-expressing cells indicates that DDR is activated to a similar level in cells harboring activated Rac1QL cells compared to H-RasV12-expressing cells. Differences of the percentages of DDR foci in the form of pATM, pS/TQ, p53BP1 and γH2AX between RasV12 or Rac1 activated cells and E.V. are statistically significant (**P* value < 0.01).

2E Schematic representation of experimental design of growth curves shown in Figure 2A and 2C is depicted.

2F Expression of dominant negative Rac1 prevents DDR activation upon H-RasV12 expression. Immunostaining as detected by confocal imaging of DDR activation marker in the form of pATM foci demonstrates that RacDN co-expression in H-RasV12 transduced cells prevents DDR activation. Scale bar: 5μm.

2G Quantification of immunostaining of DDR foci markers in the form of pATM shown in panel 2D. Differences of the percentages of DDR foci between H-RasV12 cells co- expressing E.V and RasDN are statistically significant (**P* value < 0.01).

2H Immunostaining as detected by wide-field imaging of DDR markers in the form of 53BP1 and γH2AX shown in E.V., RacDN, H-RasV12 and RacDN in H-RasV12 co-transduced cells. Scale bar: 60μm.

Supplementary Figure 3

Nox4 mRNA levels increase upon oncogenes activation and its expression is sufficient to drive hyperproliferation, DDR activation and proliferative arrest.

3A qRT-PCR analyses of E.V.-, H-RasV12- and Rac1QL-transduced cells performed at day 3 of the growth curve displayed in Figure 2A show that Nox4 mRNA levels are induced upon either oncogenes expression. (*P value < 0.01).

3B NADPH oxidase involves the conversion of NADPH to NADP+ and its activity can be evaluated by calculating the ratio of relative amounts of these species. Levels of NADP+ and NADPH in shp53 E.V. and shp53 Ras activated cells are shown. (*P value < 0.01).

3C BrdU incorporation rates of cells shown in Figure 3B. (*P value < 0.01).

3D Experimental scheme of the growth curve shown in Figure 3B is depicted.

3E Nox4 overexpression in NHFs increases ROS production as detected by DHE live cell imaging. Scale bar: 40μm.

3F Quantification of DHE signal as in panel S3D. Differences are statistically significant (*P value < 0.01).

3G qRT-PCR analyses of stable knock down of Nox4 mRNA levels in empty vector (E.V.)- and H-RasV12-expressing cells. Differences are statistically significant (*P value < 0.01).

3H Upper panel: NHFs were transduced with either E.V.- or H-RasV12-expressing lentiviruses and monitored for growth. Lower panel: NHFs were transduced using either shRNA against Nox4 gene (shNox4) together with shE.V. or H-RasV12-expressing lentiviruses.

3I Stable knockdown of Nox4 prevents H-RasV12-induced ROS accumulation, as detected by DHE live imaging and its quantification. Differences are statistically significant (*P value < 0.01). Scale bar: 40μm.

3J Quantification of immunostaining of cells at the day 5 of the growth curves shown in 3E indicates that stable knockdown of Nox4 reduces DDR activation in the form of 53BP1 and γH2AX nuclear foci in H-RasV12 cells.γH2AX foci between RasV12 with respect to shNox4/RasV12 are statistically significant (*P value < 0.01).

3K Senescence-associated beta galactoside assays show that stable knockdown of Nox4 in H-RasV12-expressing cells abolishes OIS establishment. Scale bar: 40μm.

3L The quantification of senescence-associated beta galactoside assays (panel 3K) shows that stable knockdown of Nox4 in H-RasV12 expressing cells prevents OIS. (*P value < 0.01).

3M Nox4 levels do not affect ionizing radiation (IR)-induced DDR signaling. Immunostaining as detected by widefield microscopy of DDR activation in the form of γH2AX nuclear foci in cells expressing E.V., Nox4, shE.V. or shNox4 2 hours post 2 gamma ray IR, show similar degrees of DDR activation. Scale bar: 60μm.

3N Quantification of immunostainings in 3M shows no significant difference of DDR

activation upon IR with respect to Nox4 expression levels in NHFs.

3O Quantification of average intensity of γH2AX nuclear foci upon IR with respect to Nox4 expression levels in NHFs shows no significant difference. The average intensity is calculated by using Image J. Arbitrary units (a.u.) are used.

Supplementary Figure 4

Nox4 inhibitors reduce ROS production and DDR foci formation in oncogenic Ras-expressing cells.

4A Quantification of DHE signal in Figure 4A. Treatment of oncogenic Ras expressing cells with Nox4 inhibitors reduces Ras-induced ROS levels statistically significantly. (**P* value < 0.01).

4B Nox4i treatment prevents H-RasV12-induced ROS accumulation, as detected by Peroxy Orange 1 (PO1) live imaging (Dickinson et al, 2010). H_2O_2- specific detection method. PO1 relies on a chemospecific boronate-to-phenol switch to respond to H_2O_2 over a panel of biologically relevant ROS with excitation at 543 nm and emission around 560 nm sensitivity to endogenously produced H_2O_2 signals. The cells were transduced with lentiviruses expressing either E.V. or Ras or Nox4 together with GFP, so that cells could be visualized during live imaging. Scale bar: 40μm.

4C Quantification of PO1 signal in S4B. (**P* value < 0.01).

4D, E Representative confocal images of immunostaining of DDR markers in the form of 53BP1 and γH2AX foci of cells treated by the vehicle (DMSO) or Nox4i either expressing H-RasV12 (panel A) or empty vector (E.V.; panel B). Scale bar: 5μm.

Supplementary Figure 5

Ras activation leads to upregulation of zebrafish Nox4 gene expression.

5A Nox4 protein has a transmembrane-spanning, carboxy-terminal binding sites for FAD and NADPH. Alignment of the sequences of *Homo sapiens, Takifugu rubripes* and *Danio rerio* zebrafish species for *Nox4* gene using Clustal OMEGA.

5B Zebrafish genome analyses reveal three potential orthologues of human Nox4. Ensembl accession numbers of the genesare designated as gene1 (ENSDARP00000105348), gene2 (ENSDARP00000104815), gene3 (ENSDARP00000110067). All three ORFs were detected by qRT-PCR 24 hours post heat-shock. All genes are expressed, and genes 1 and 3 are induced upon H-RasV12 induction.

Supplementary Figure 6

Nox4 gene expression is upregulated in various cancer types.

6A "Secondary antibody only" control experiment shows negative staining on mouse

pancreatic tissue. Scale bar: 100μm.

6B Pancreatic islets in normal pancreas known to express NOX4 serve as a positive control for immunostaining (arrow). Normal acinar tissue is NOX4 negative, as described in the main text. Scale bar: 100μm.

6C, D Nox4 antibody specificity is checked by performing immunofluorescence staining on NHFs transduced with either E.V. or H-RasV12 or Nox4 expressing lentiviruses or two distinct lenti shRNAs against Nox4. Stable knockdowns of Nox4 by two distinct shRNA-expressing lentiviruses diminished staining with the Nox4 antibody used. Scale bar: 60μm. Reduced expression levels of Nox4 were confirmed by qRT.PCR**.**

6E Unfixed frozen OCT sections of Pdx1-cre; LSL-KRasG12D mouse sections at various stages (no cre, 7 and 9 months post cre induction) were probed for mRNA levels of Nox4 and Nqo1 (a Nrf2 target gene) by qRT.PCR.

6F Parallel OCT sections as in panel 6E were probed for ROS level by DHE probe and imaged. Scale bar: 100μm.

6G Quantification of DHE signal of panel 6F. Differences are statistically significant (*P value < 0.01).

6H Paraffin-embedded parallel sections of Figure 6A were probed for Nqo1 on sections of WT and p48-Cre; LSL-KrasG12D mouse pancreata. Scale bars are indicated.

6I Paraffin-embedded parallel sections of Figure 6B were probed for Nqo1 on sections of human normal pancreas, early panIN and late invasive tumors (pancreatic adenocarcinoma). Scale bars are indicated.

6J Summary of Nox 1-5 genes overexpression in cancerous versus normal human tissue analyses according to Oncomine datasets shows that Nox4 gene is upregulated in many cancers studied. Colours indicate whether the fold change is among the 1%, 5% or 10% most upregulated (red) or downregulated (blue) genes. The white box corresponds to not in set of 10% most up-/down-regulated genes.

Supplementary Figure 7

H-RasV12 expressing, DDR-deficient cells depend on Nox4 for proliferation.

7A, B ROS production as detected by live imaging with DHE probe in BJhTERT, BJ ELR, BJ hTERTshp53 and BJ hTERTshp53 Ras cells. Scale bar: 60μm.

7C, D ROS generated in cells shown in S7A, B were quantified using Image J. In addition, quantification of ROS levels were performed using CM-H_2DCFDA fluorescent ROS probe. Quantification of ROS accumulation detected by DHE probe and CM- H_2DCFDA fluorescent ROS probe were at comparable levels (Fore details, see Materials & Methods section).

7E, F Both BJ hTERT and BJELR cells were cultured in the presence of NAC (or NAA) or either Nox4 inhibitors (or DMSO) at 5µM concentration for six consecutive days. The fold increase in cell numbers for 6 days since day 1 (panel E), and BrdU incorporation rates for day 6 (panel F) are shown. Error bars indicate s.e.m. ($n \geq 3$), and differences between treatments NAA versus NAC and DMSO versus Nox4is are statistically significant (**P* value < 0.01 and ***P* value <0.05) throughout the figure where stated.

7G, H BJhTERTshp53andBJhTERTshp53Rascellsweretreatedasdescribedabove. The growth fold increase in cell numbers (panel G) and BrdU incorporation rates (panel H) show that oncogenic Ras expression is sufficient to confer ROS depletion sensitivity.

7I PCNA, CDC6 and MCM6 mRNA levels as detected by qRT-PCR in BJELR cells treated with DMSO, Nox4i1 or Nox4i2.

7J IL1 alpha, Il6 and IL8 mRNA levels as detected by qRT-PCR in BJELR cells treated with DMSO, Nox4i1 or Nox4i2.

7K Quantification of CM-H_2DCFDA signal in BJELR cells treated with DMSO, Nox4i1 or Nox4i2.

7L PCNA, CDC6 and MCM6 mRNA levels as detected by qRT-PCR in shp53 Ras cells treated with DMSO, Nox4i1 or Nox4i2.

7M IL1 alpha, Il6 and IL8 mRNA levels as detected by qRT-PCR in shp53 Ras treated with DMSO, Nox4i1 or Nox4i2.

7N Quantification of CM-H_2DCFDA signal in shp53 Ras cells treated with DMSO, Nox4i1 or Nox4i2.

7O Whole cell lysates of PANC1 cells treated with DMSO or gemcitabine alone or gemcitabine in combination with Nox4i1 or Nox4i2, or Nox4i1 or Nox4i2 alone as in Figure 7B were probed for caspase 3 activation by western blotting. Vinculin was used as a loading control.

Supplementary Figure 1, Ogrunc et al.

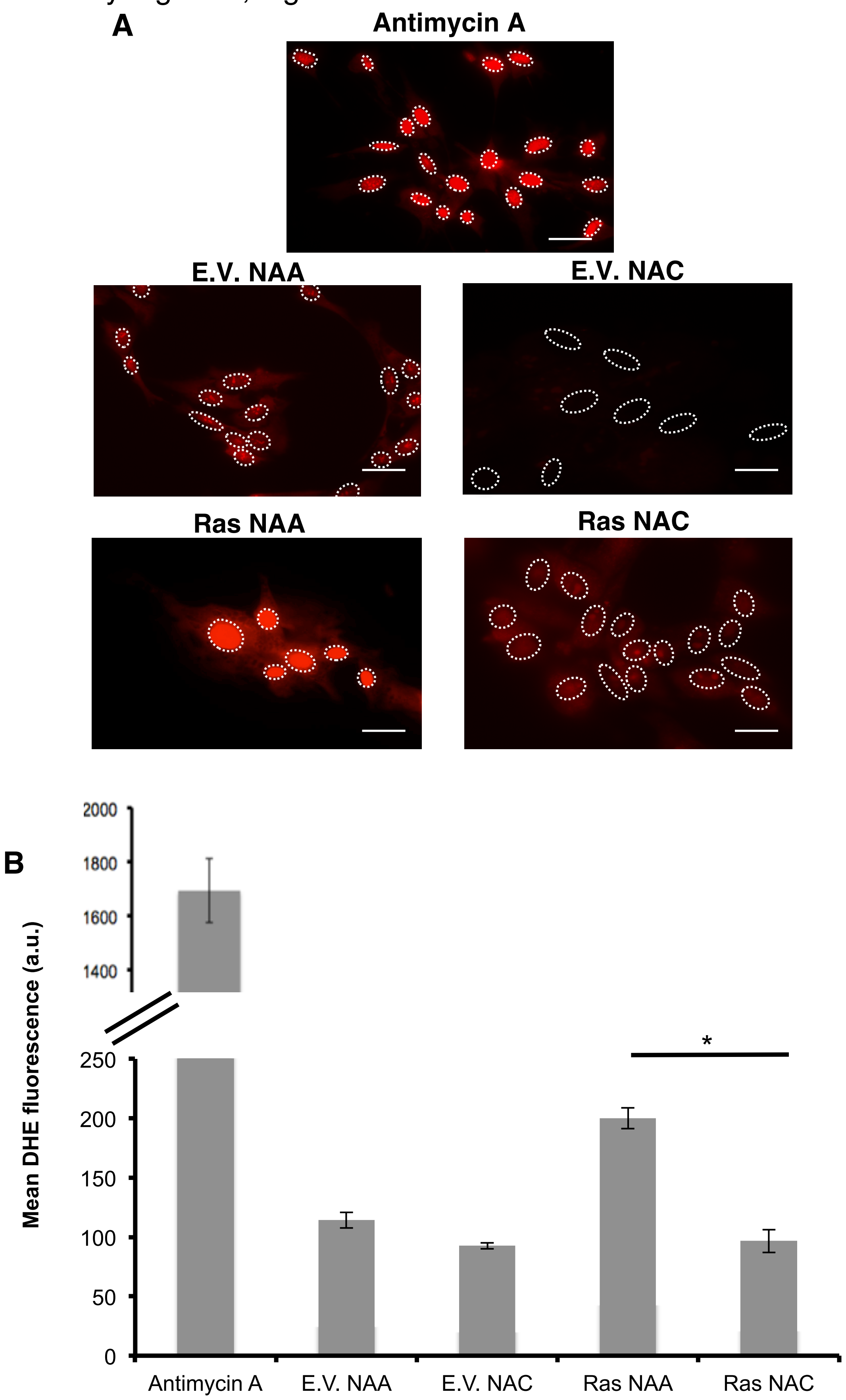

Supplementary Figure 1, Ogrunc et al.

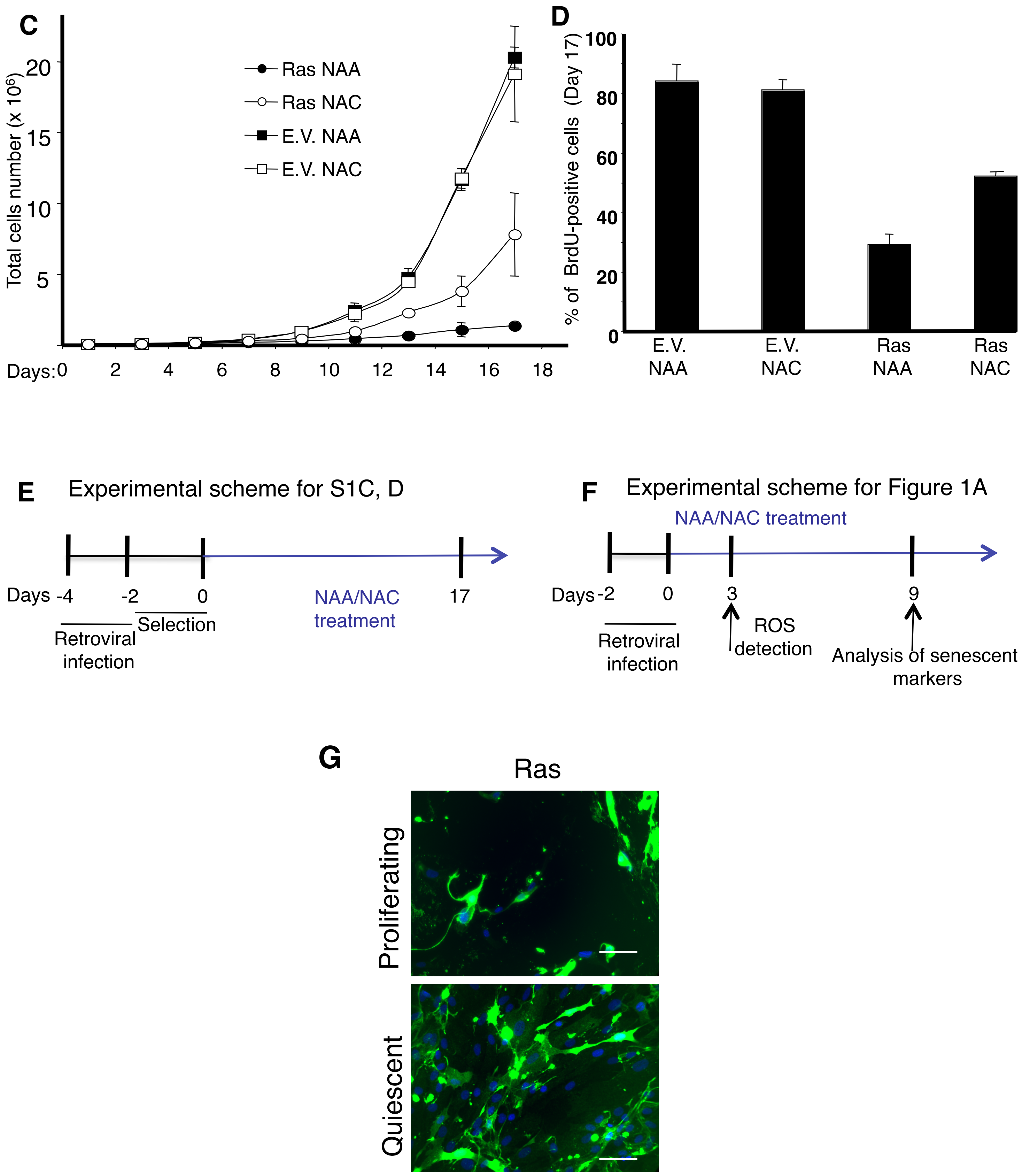

Supplementary Figure 1, Ogrunc et al.

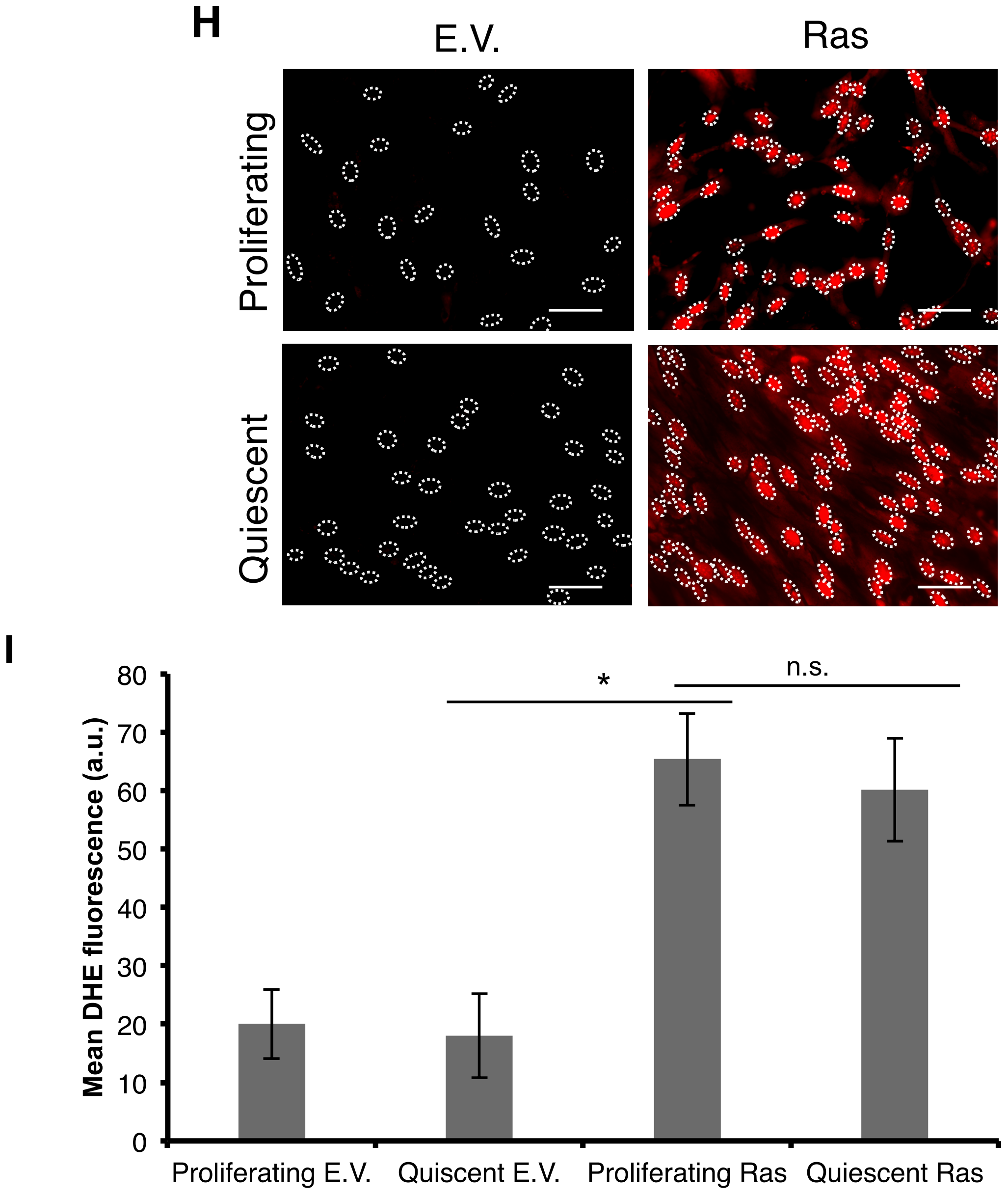

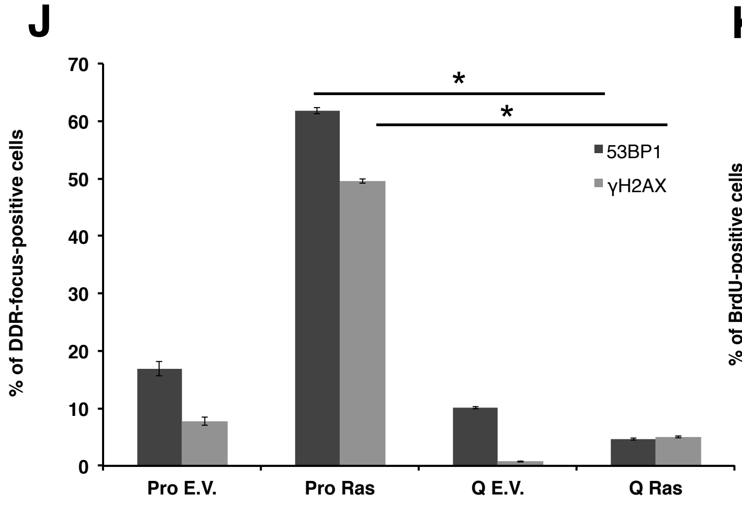

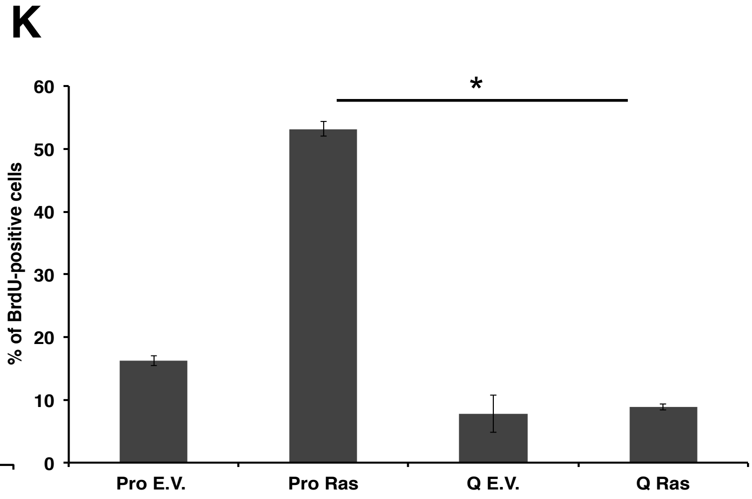

Supplementary Figure 2, Ogrunc et al.

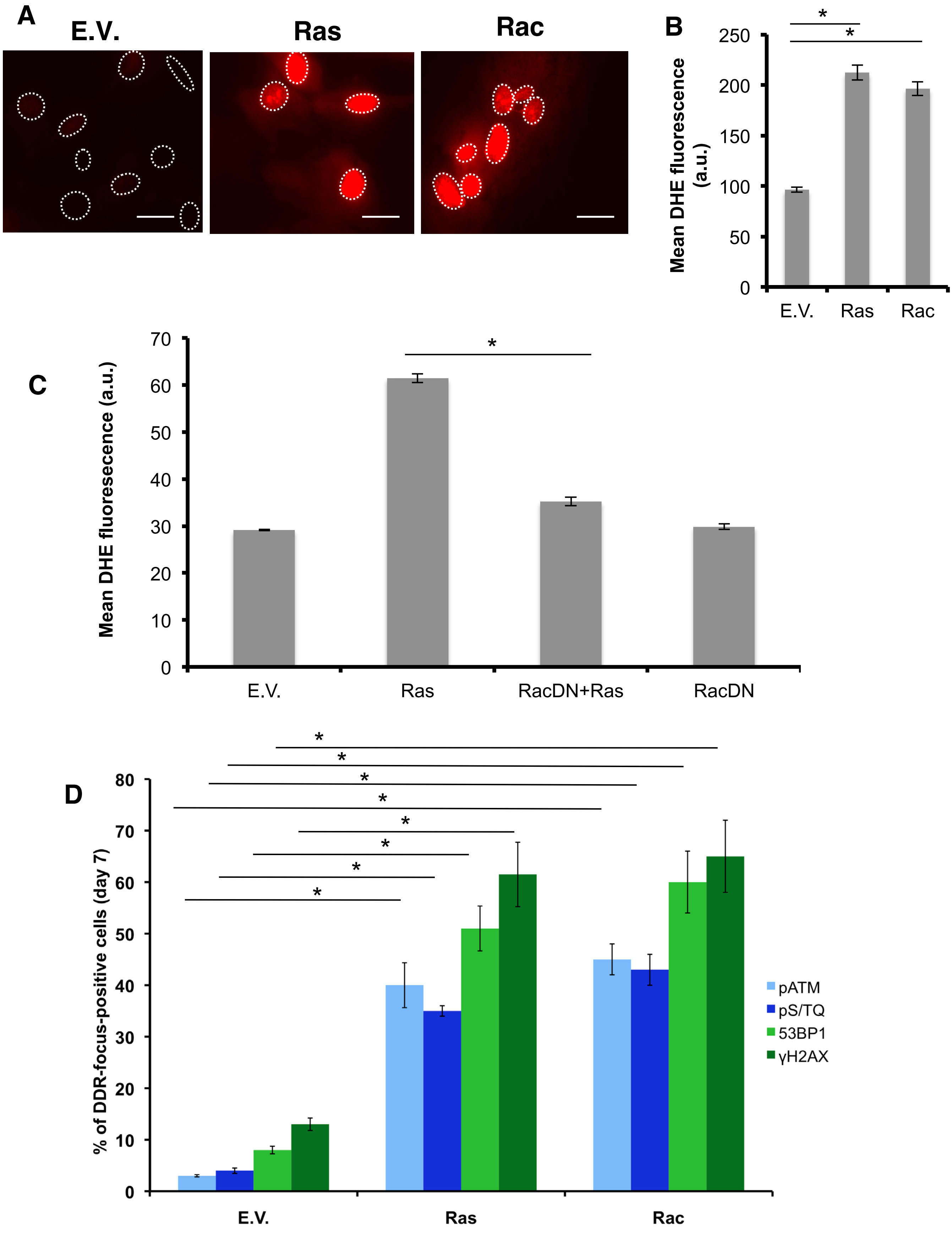

Supplementary Figure 2, Ogrunc et al.

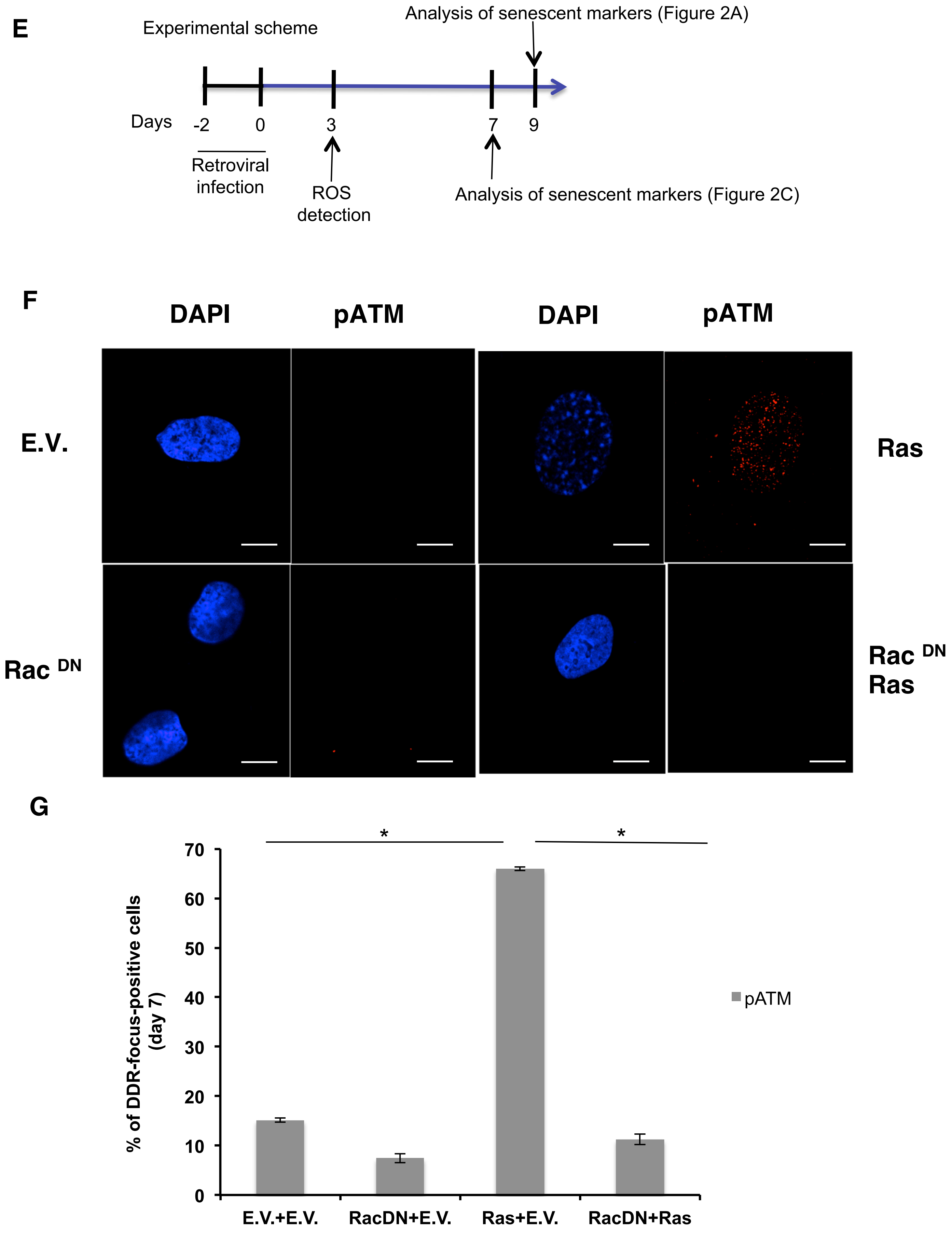

Supplementary Figure 2, Ogrunc et al.

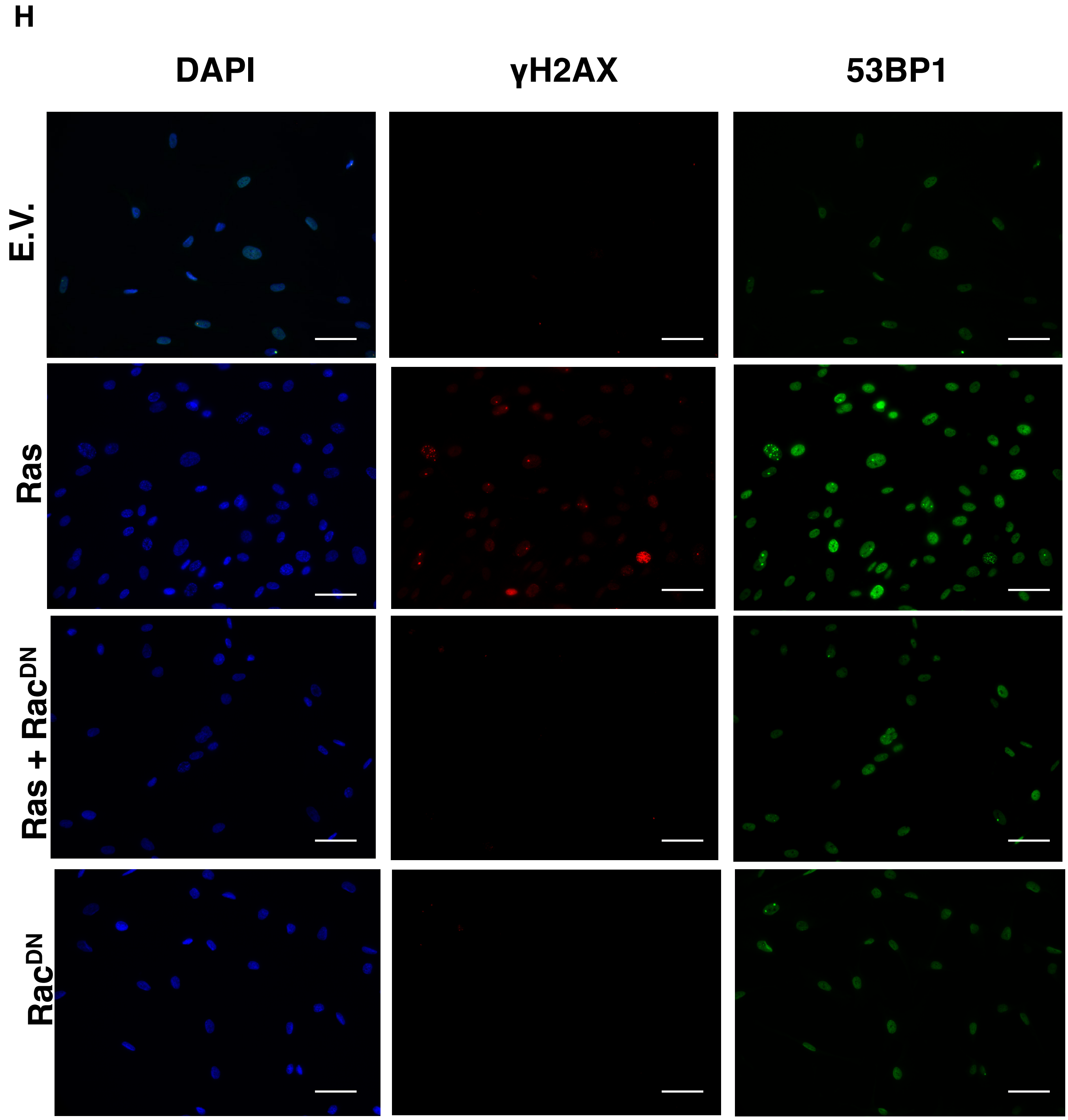

Supplementary 3, Ogrunc et al.

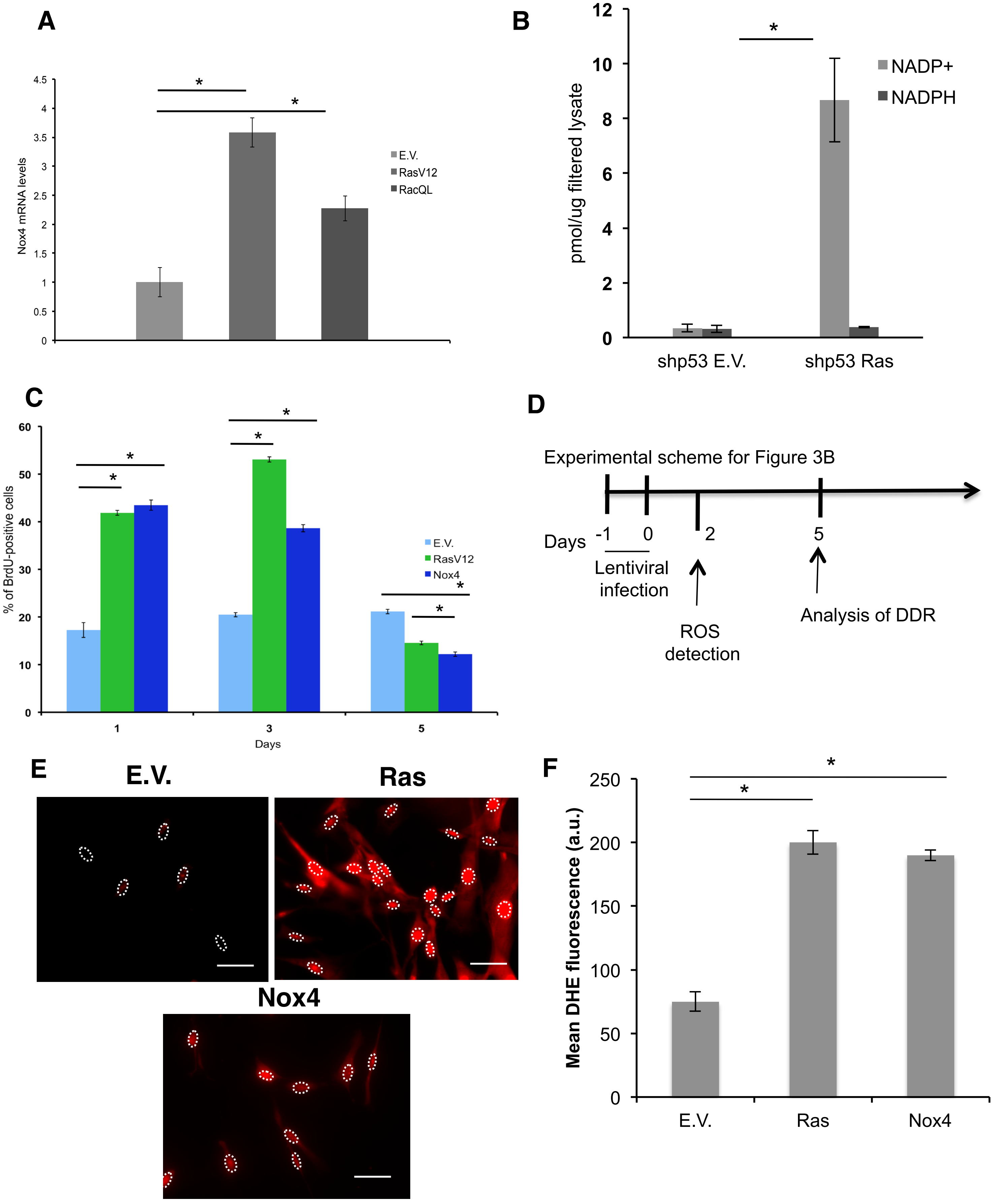

Supplementary 3, Ogrunc et al.

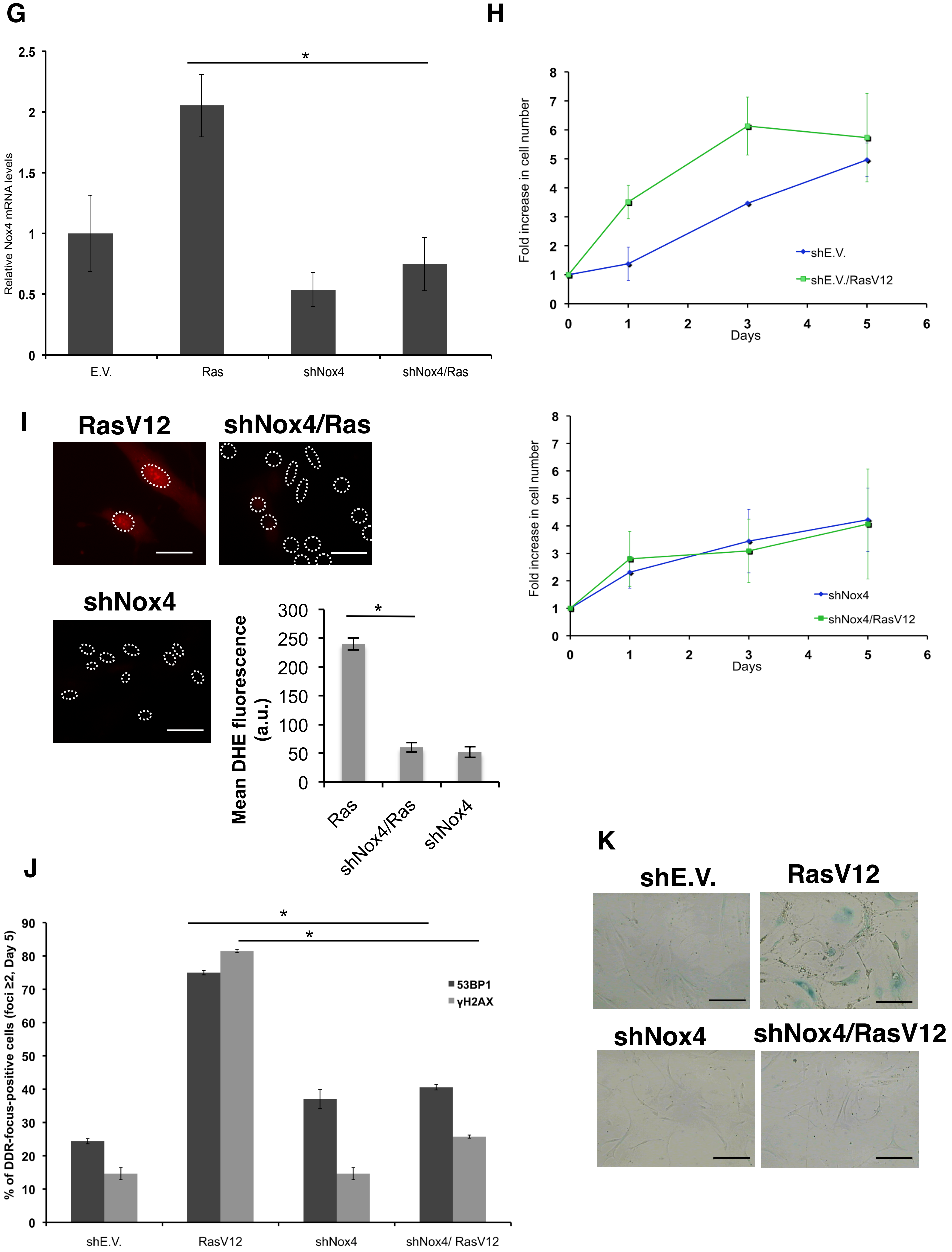

Supplementary 3, Ogrunc et al

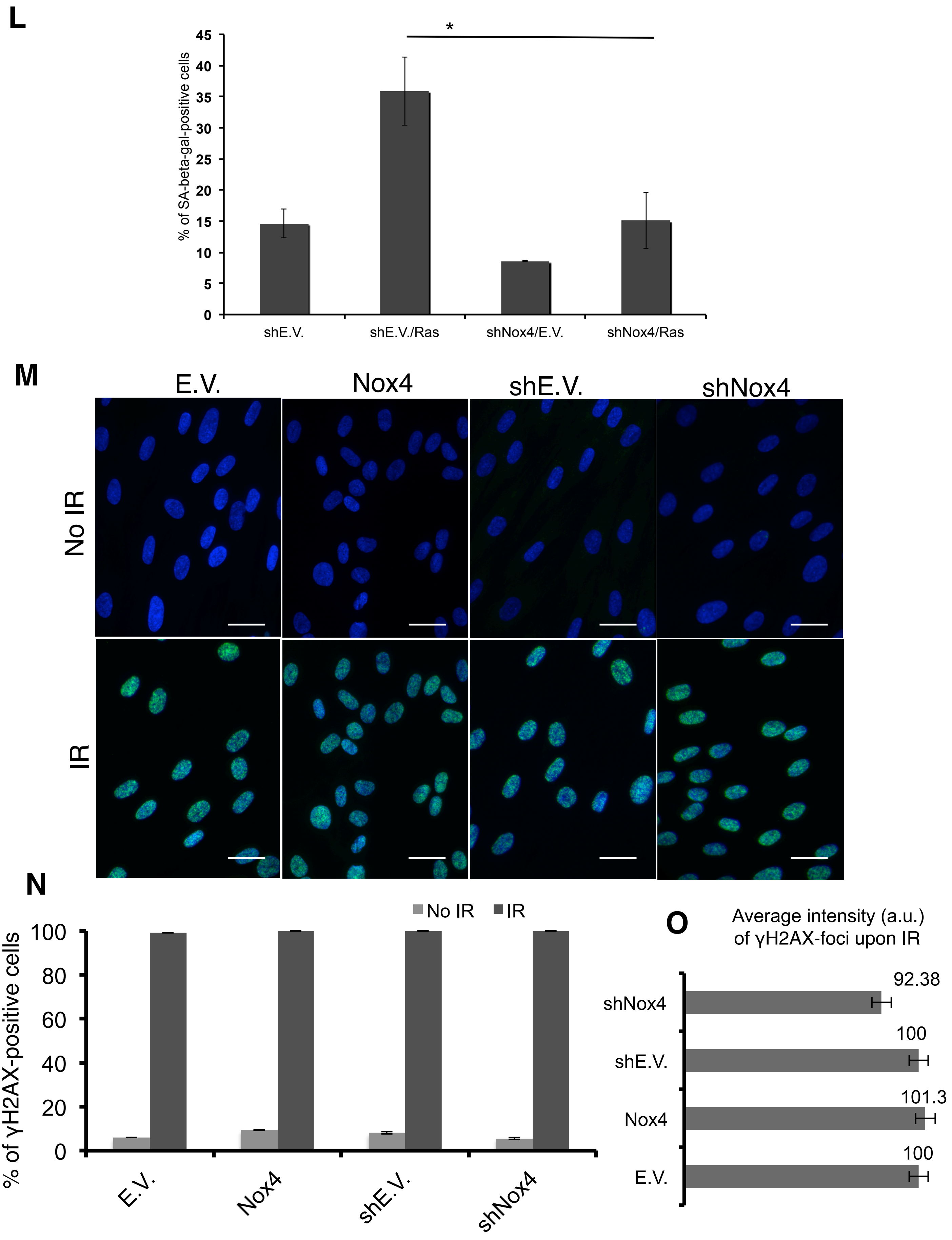

Supplementary Figure 4, Ogrunc et al

A

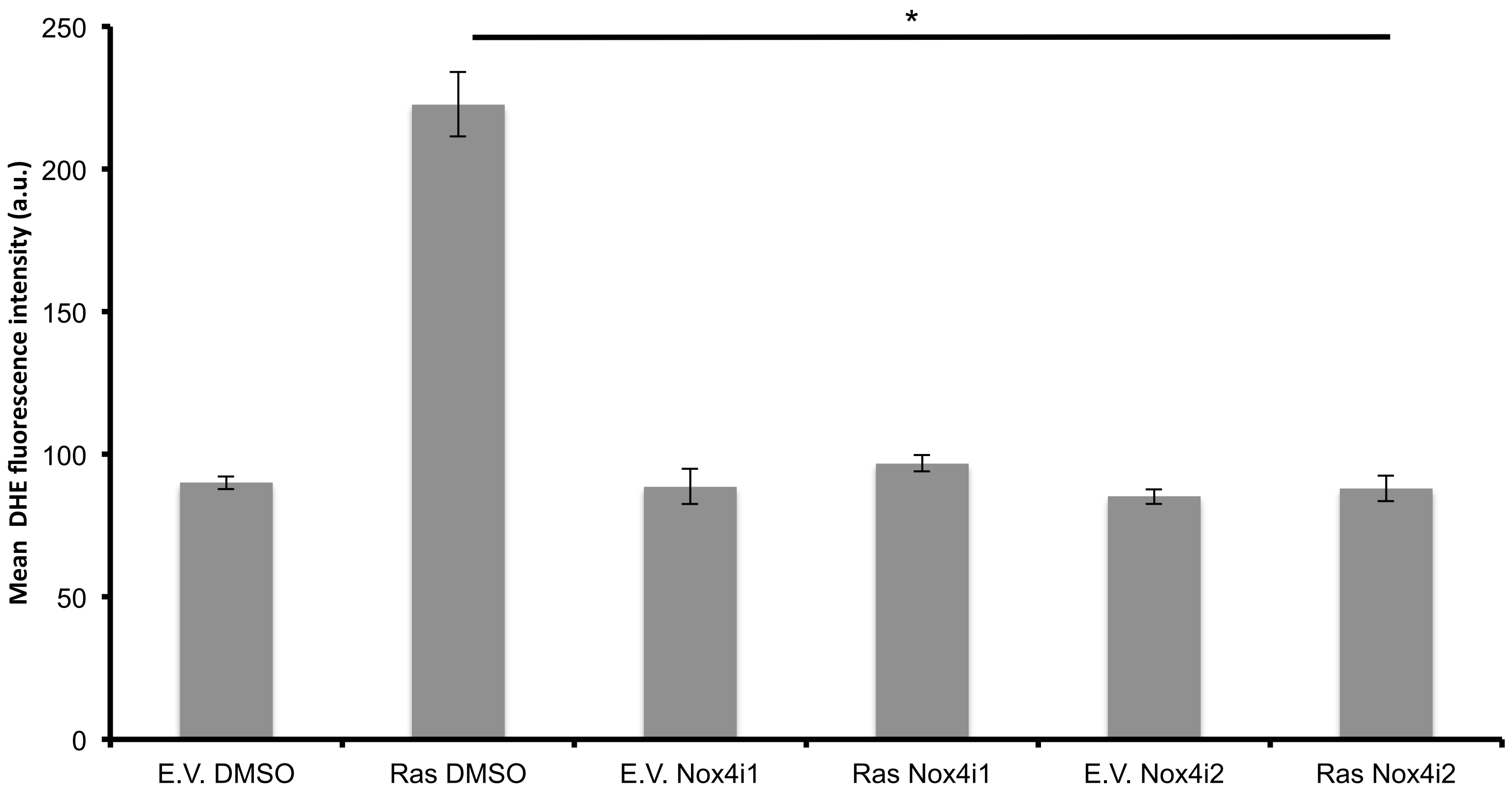

Supplementary Figure 4, Ogrunc et al

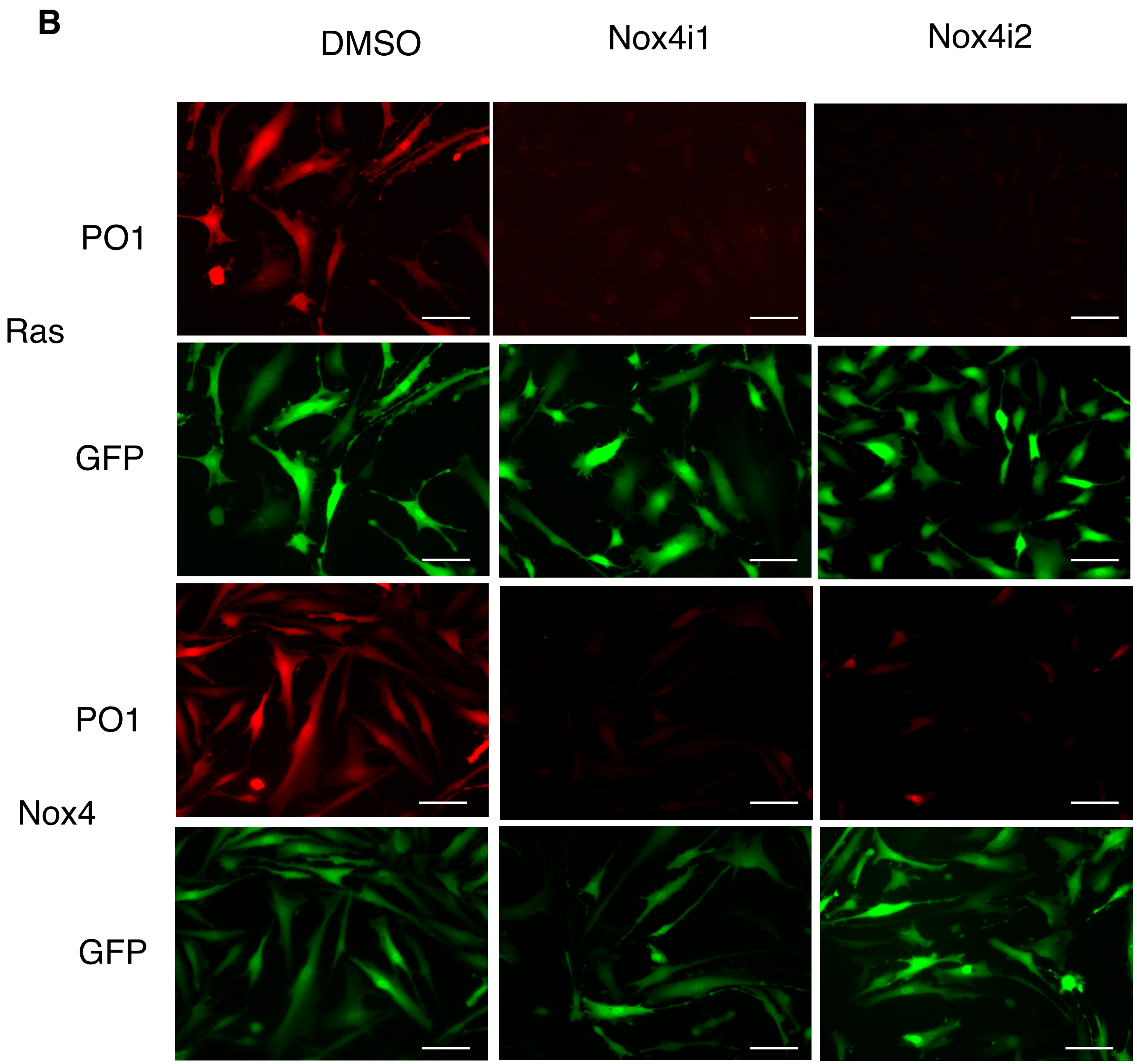

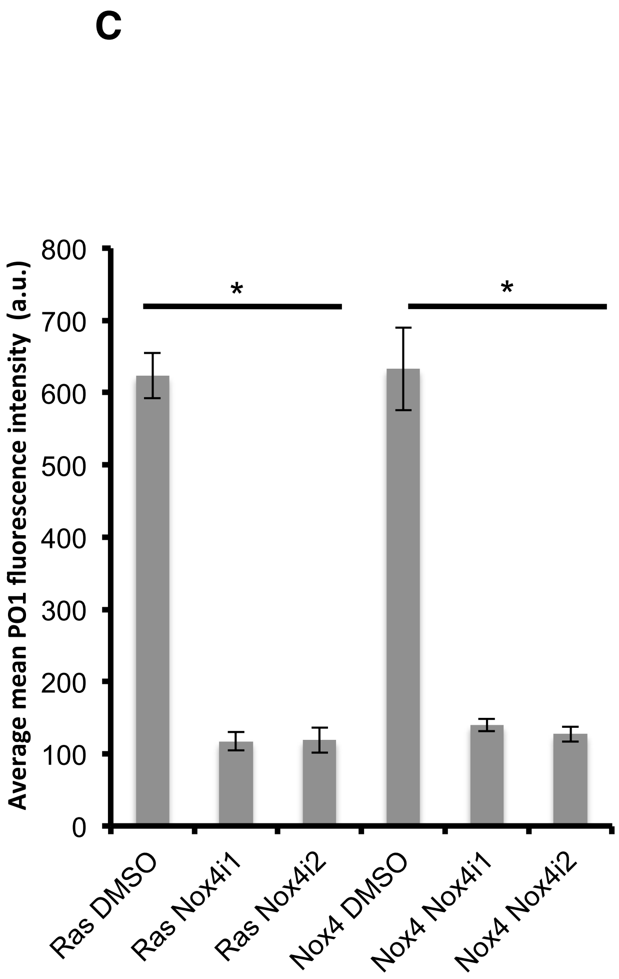

Supplementary Figure 4, Ogrunc et al

B continued

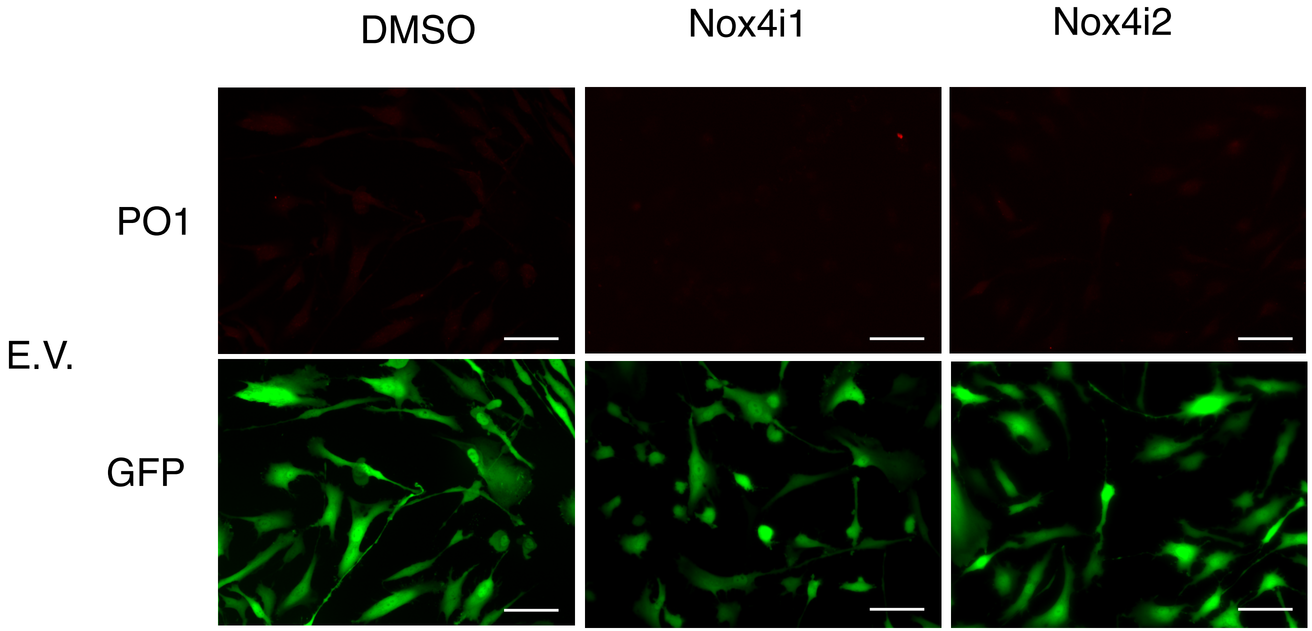

Supplementary Figure 4, Ogrunc et al

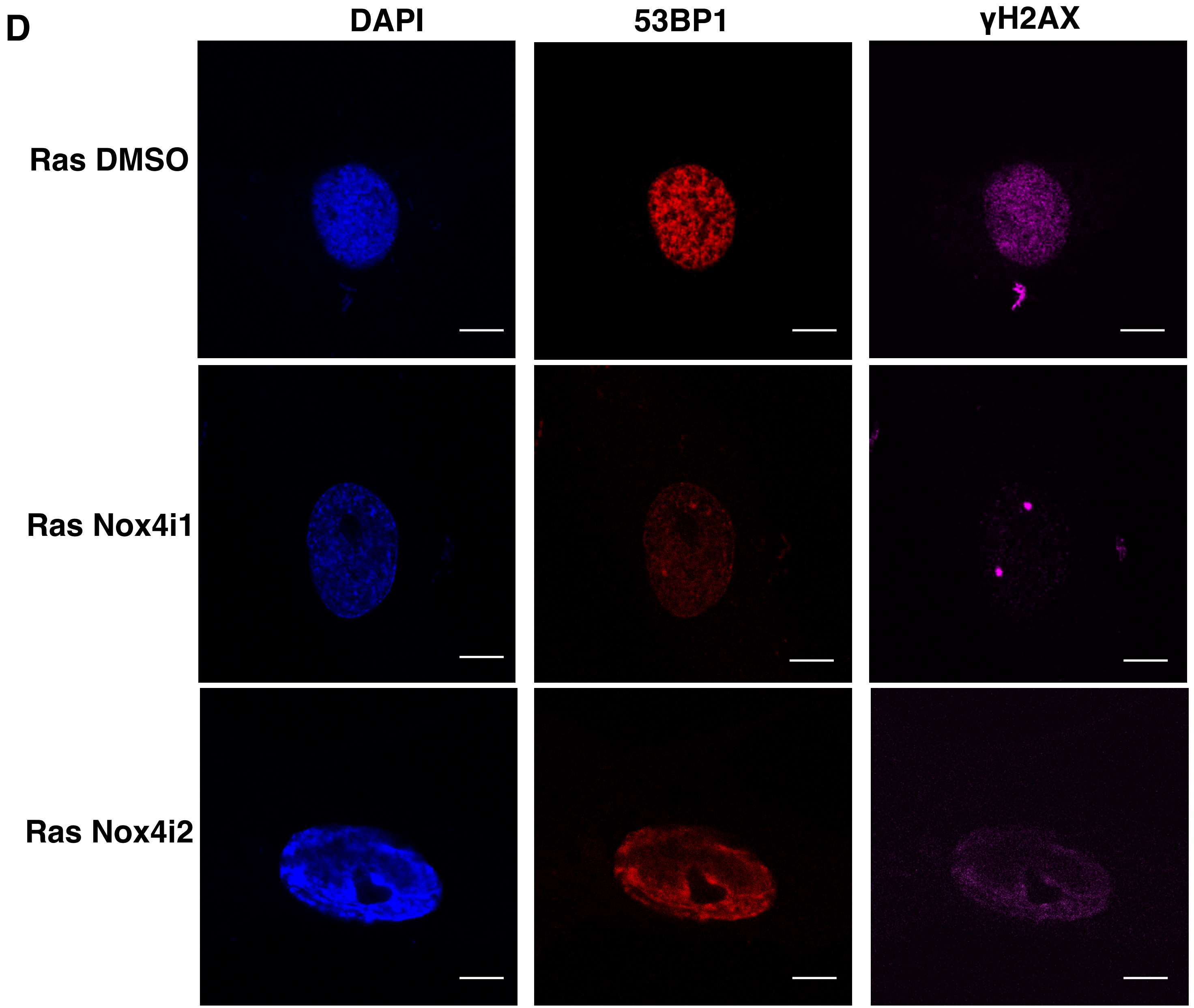

Supplementary Figure 4, Ogrunc et al

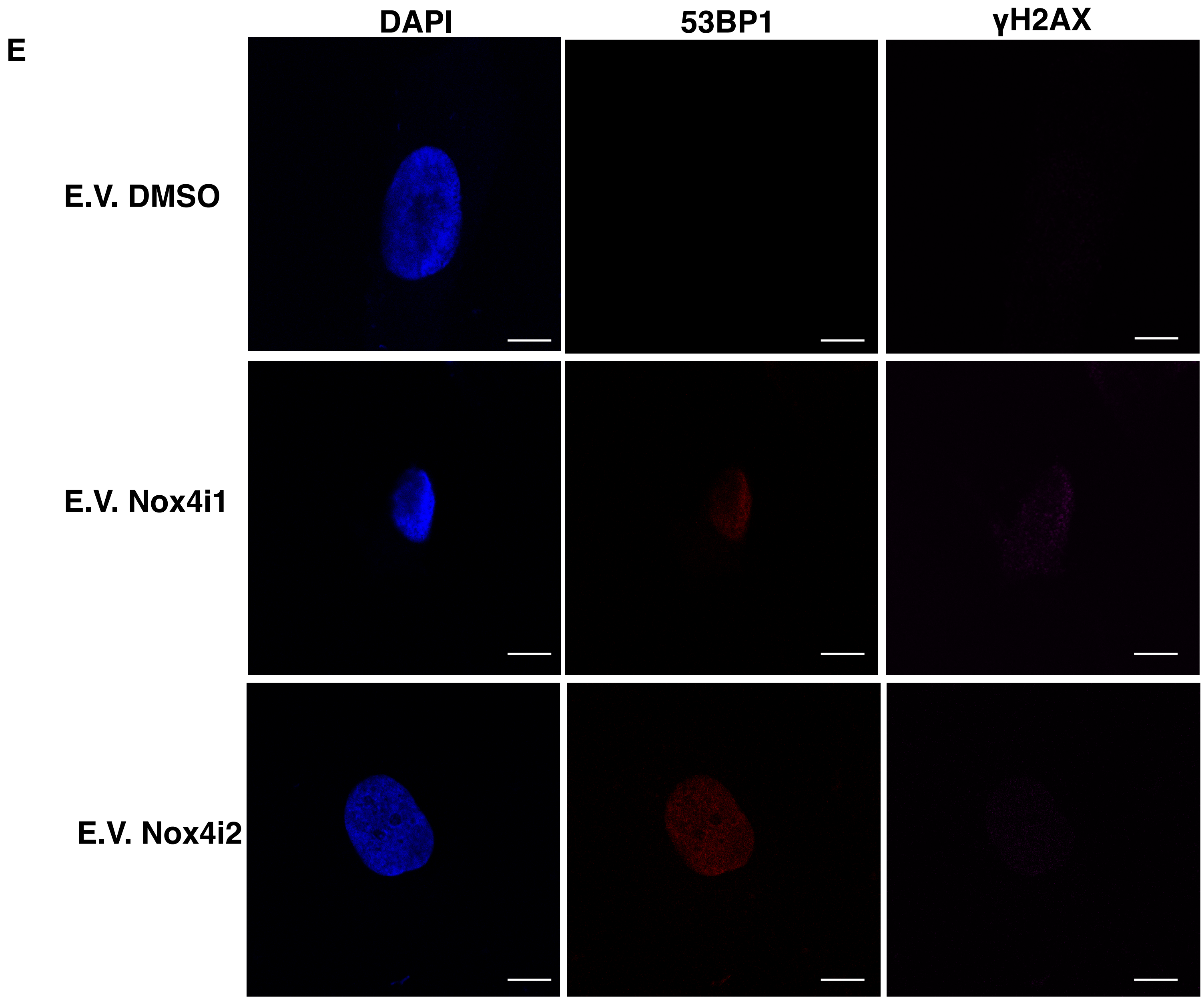

Supplementary 5, Ogrunc et al.

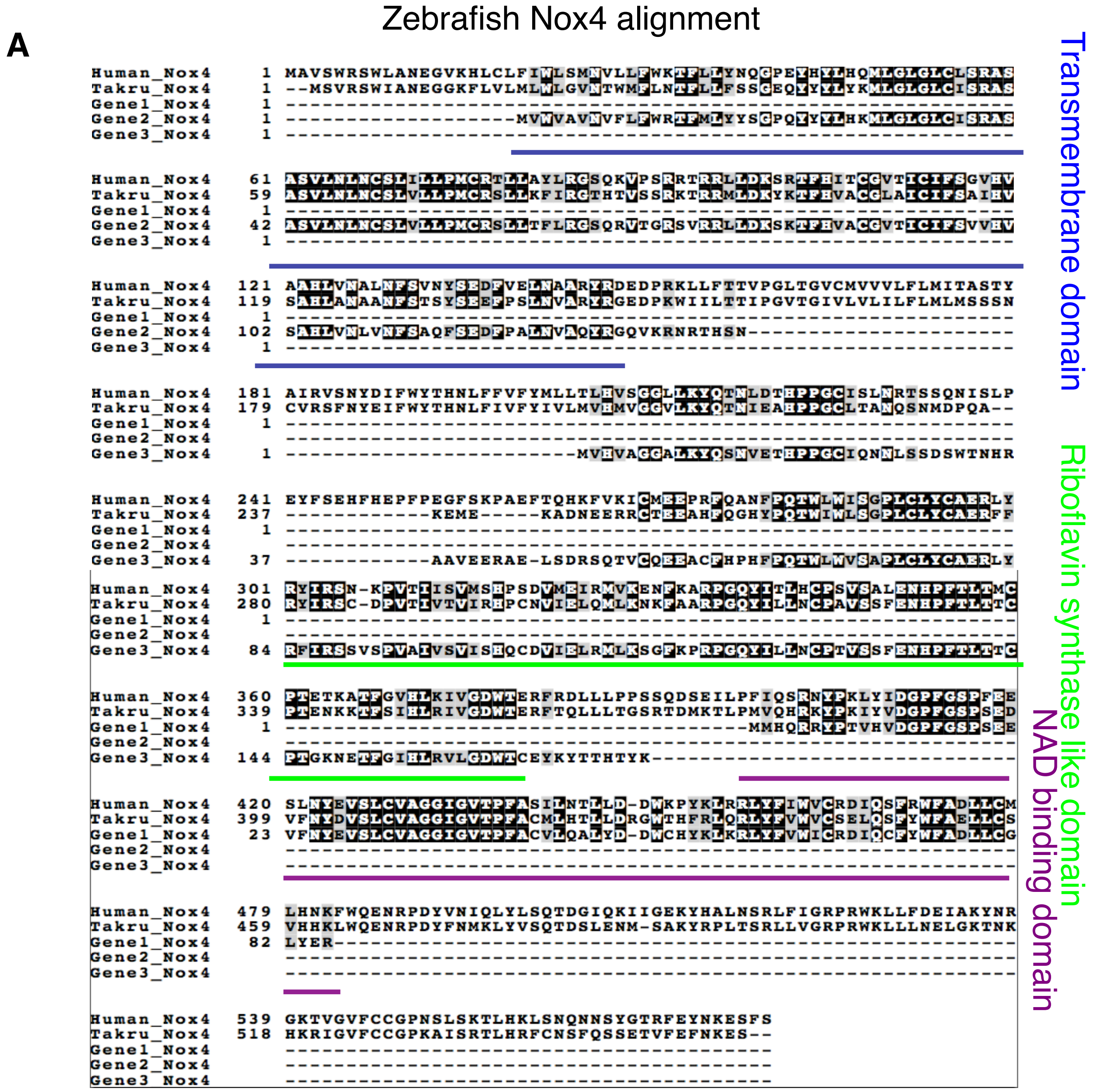

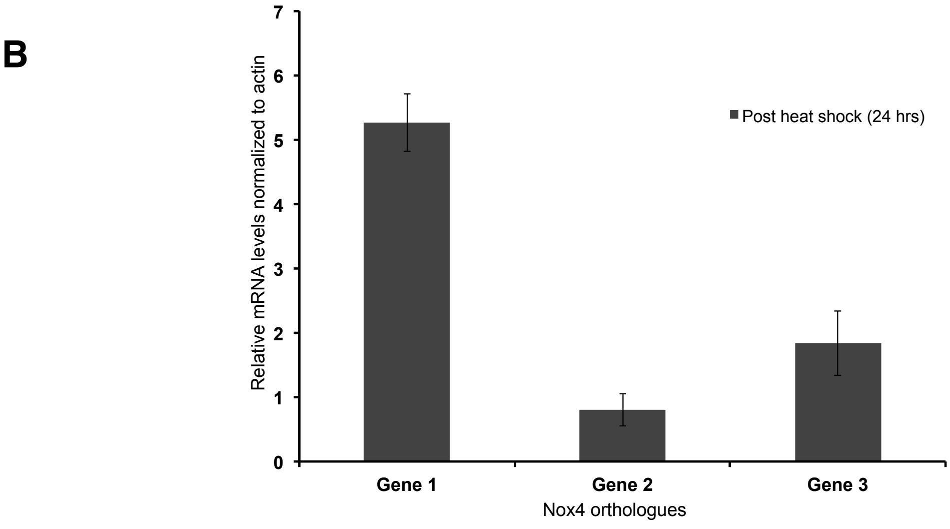

Supplementary 6, Ogrunc et al.

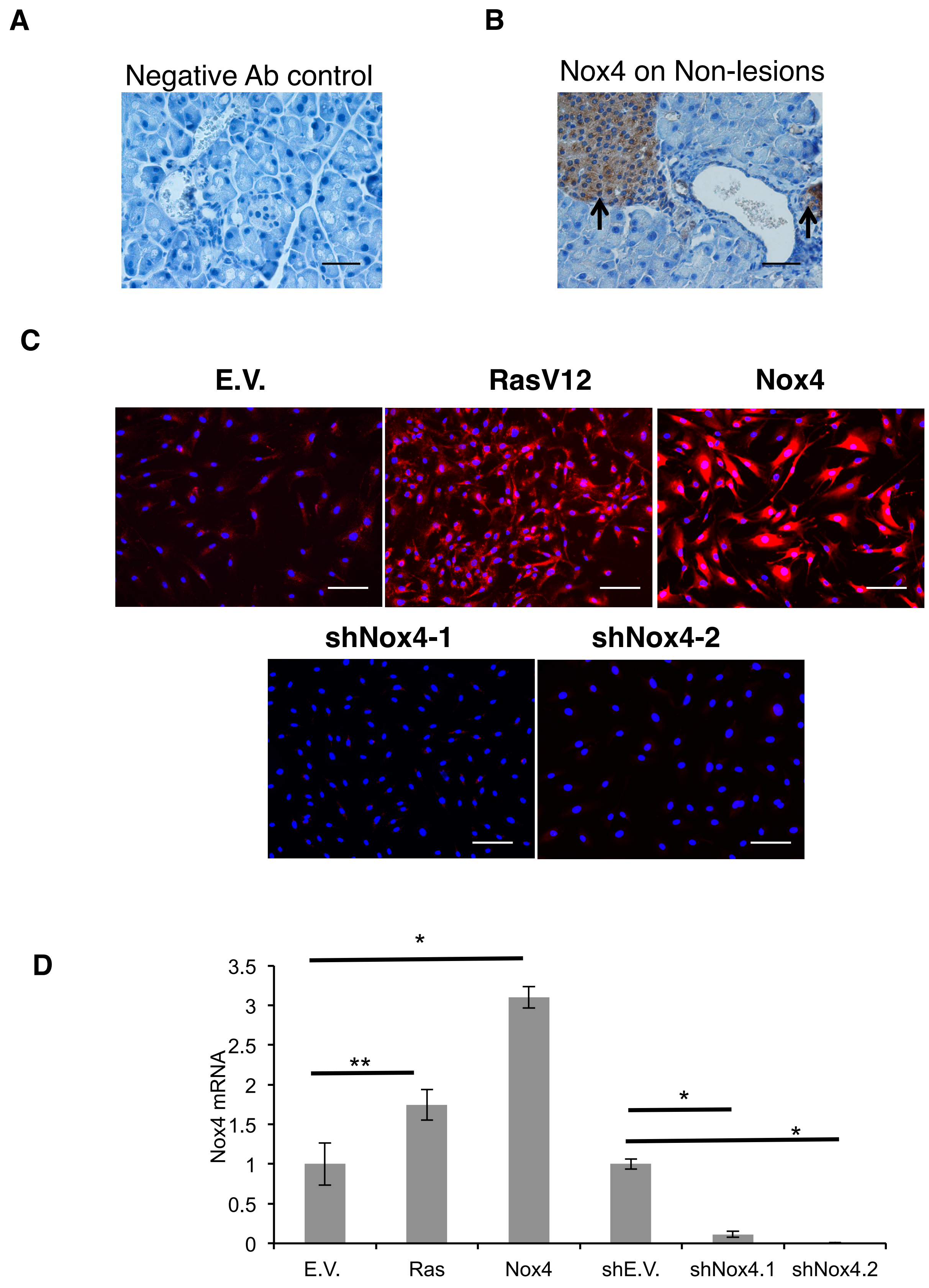

Supplementary 6, Ogrunc et al.

E

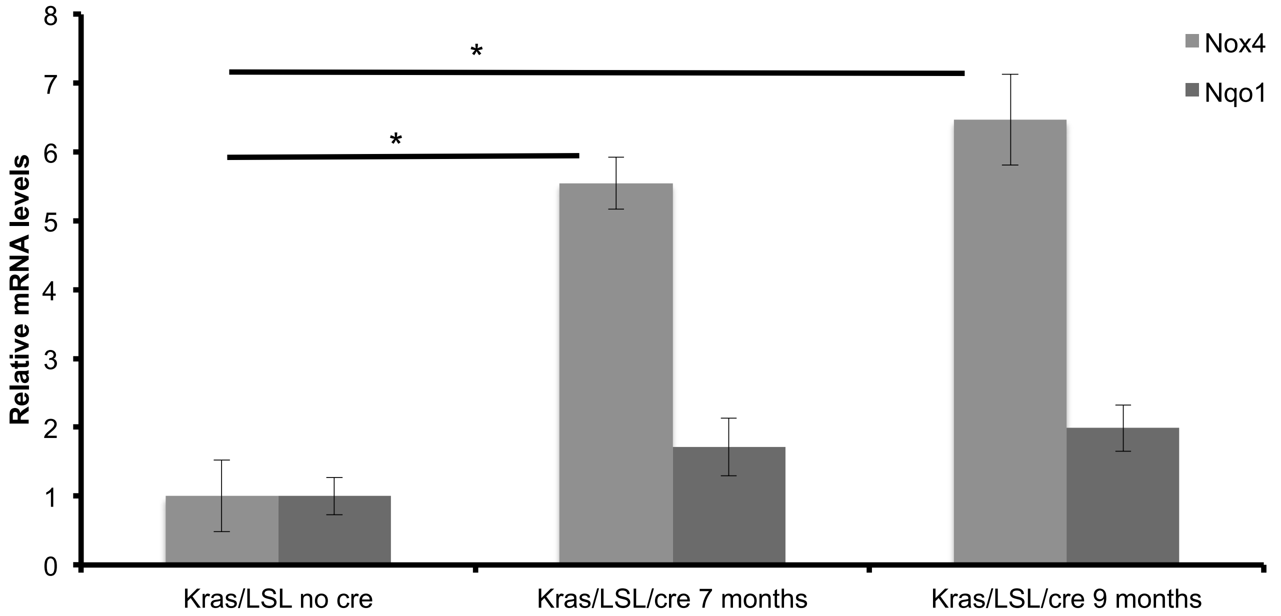

F

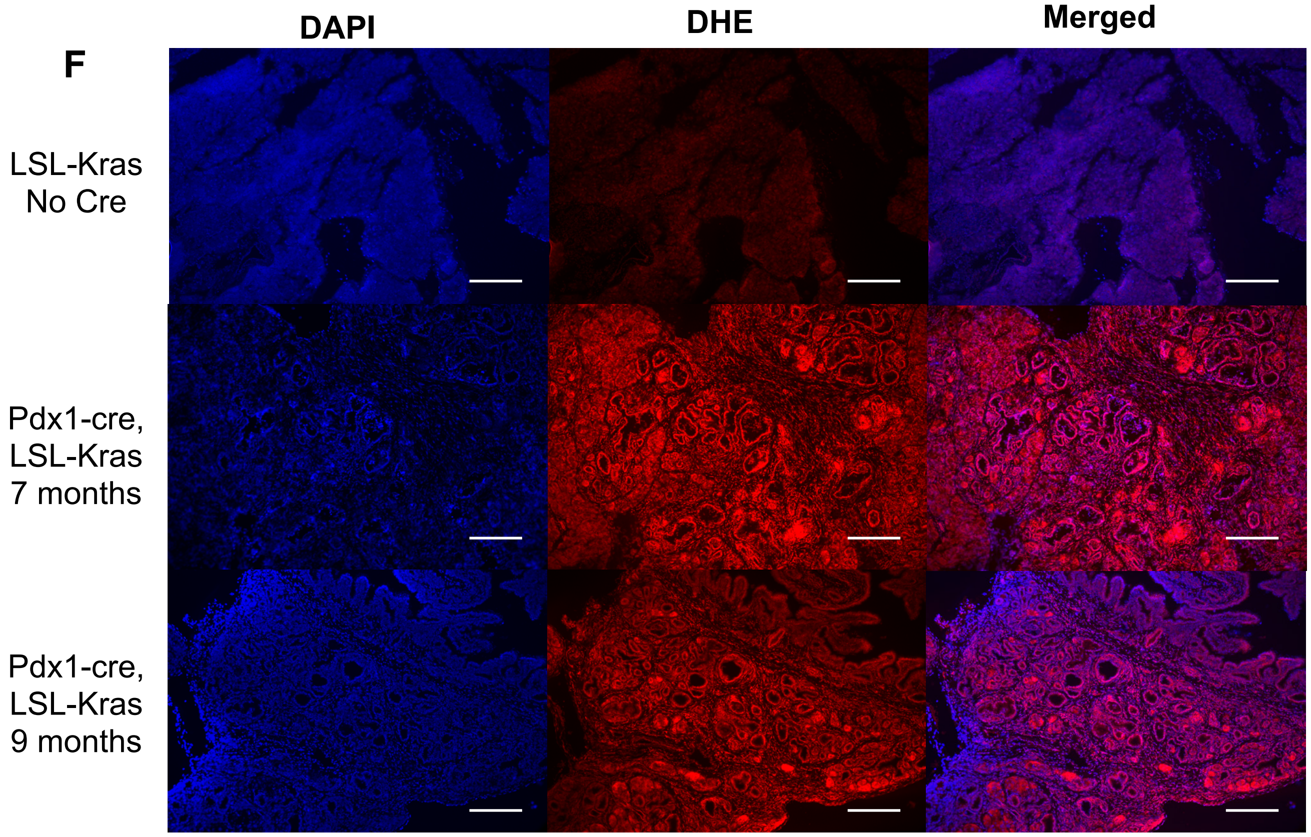

Supplementary 6, Ogrunc et al.

G

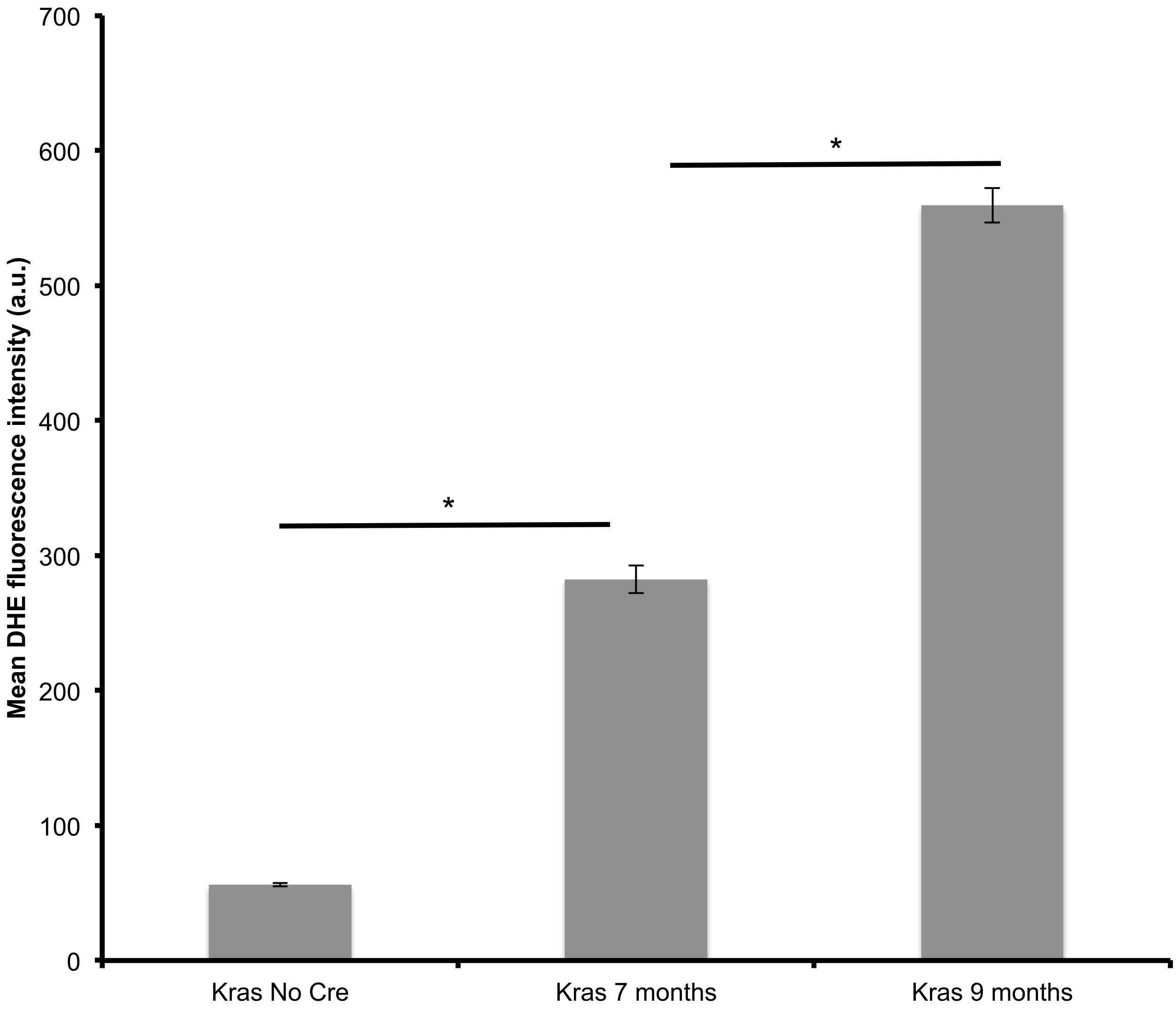

Supplementary 6, Ogrunc et al.

H

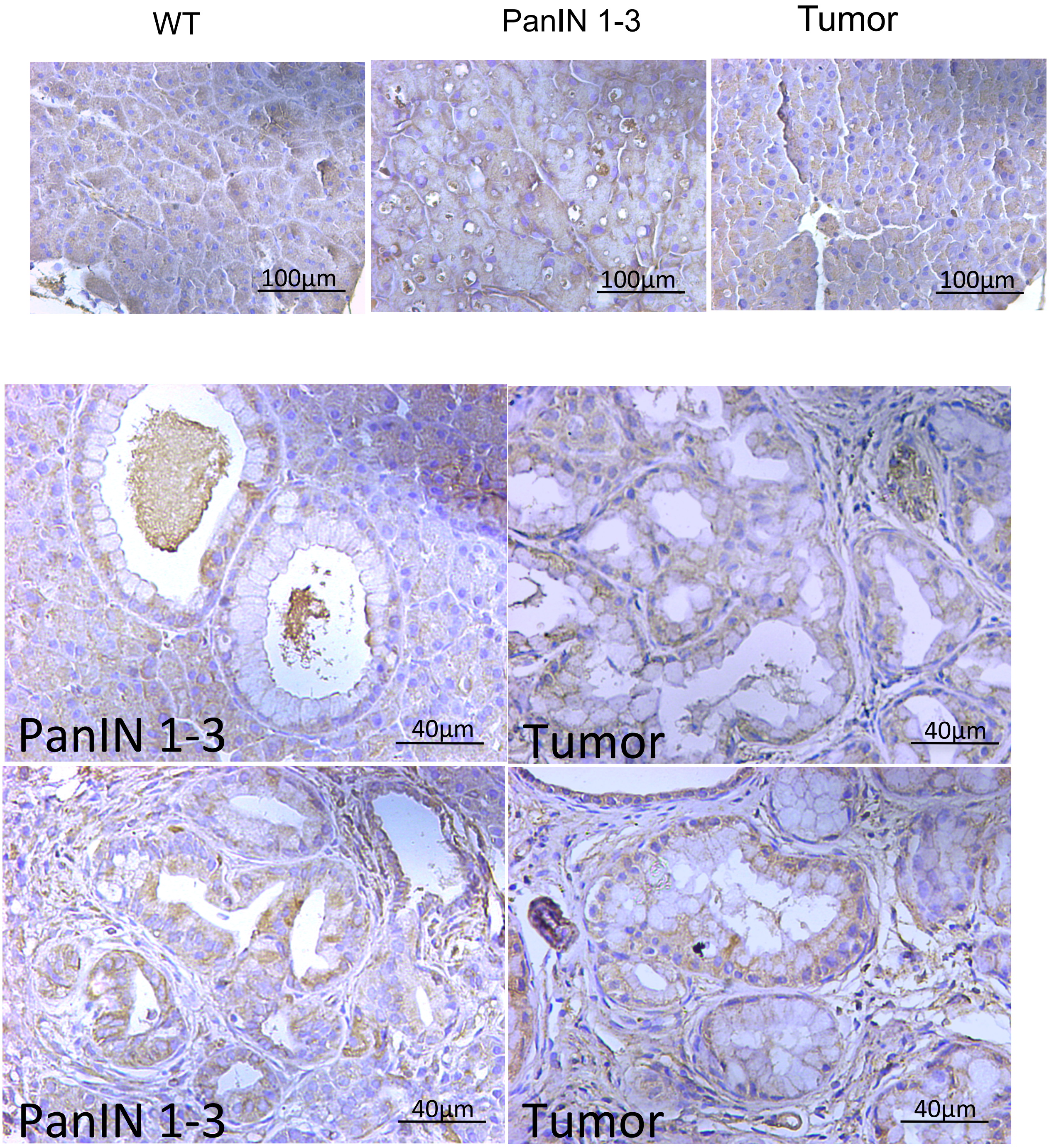

Supplementary 6, Ogrunc et al.

I

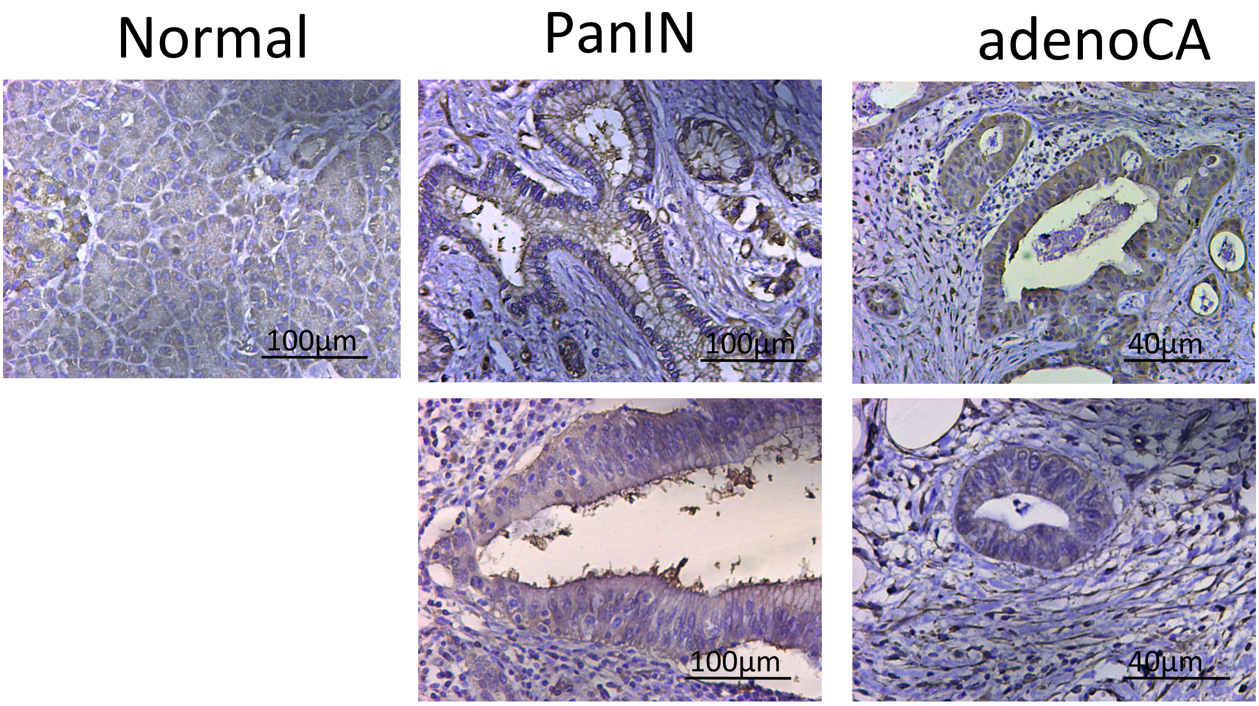

Supplementary 6, Ogrunc et al.

J

Cancer types of the Cancer vs Normal Analysis	**Nox1**	**Nox2**	**Nox3**	**Nox4**	**Nox5**
Bladder cancer					
Brain and CNS cancer					
Breast cancer					
Cervical cancer					
Colorectal cancer					
Esophageal cancer					
Gastric cancer					
Head and Neck cancer					
Kidney cancer					
Leukemia					
Liver cancer					
Lung cancer					
Lymphoma					
Melanoma					
Ovarian cancer					
Pancreatic cancer					
Prostate cancer					
Sarcoma					
Other cancers					

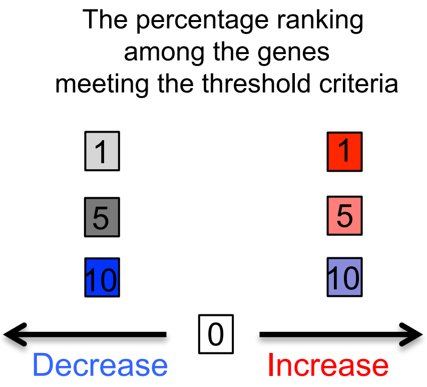

Supplementary 7, Ogrunc et al

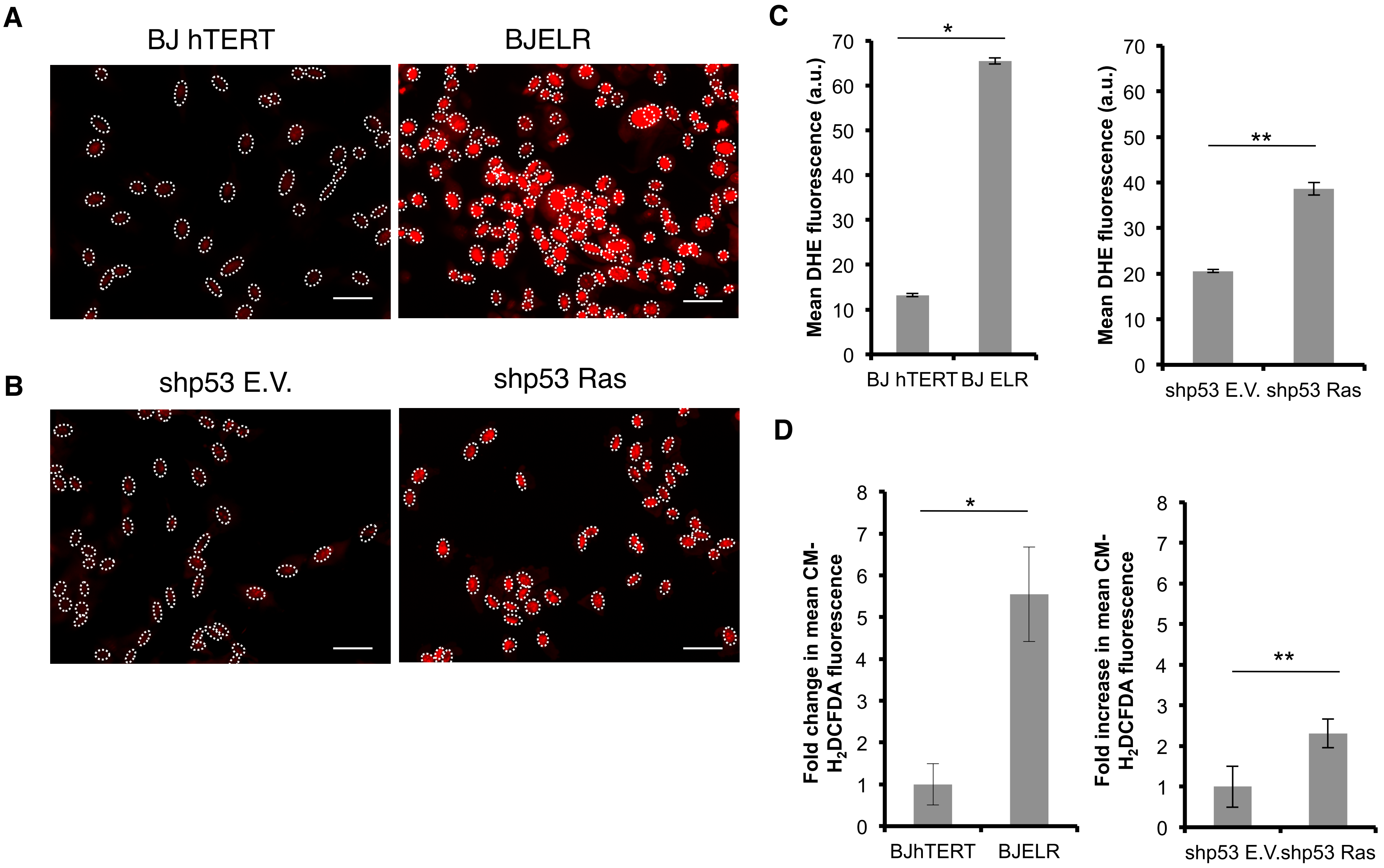

Supplementary 7, Ogrunc et al

E

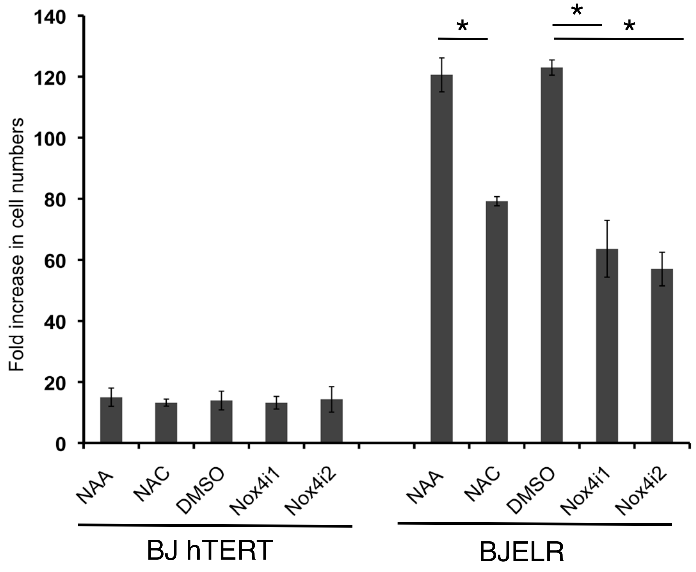

F

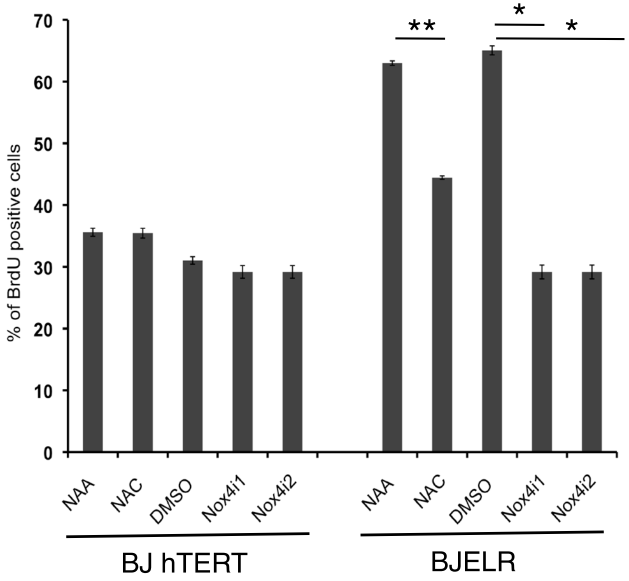

G

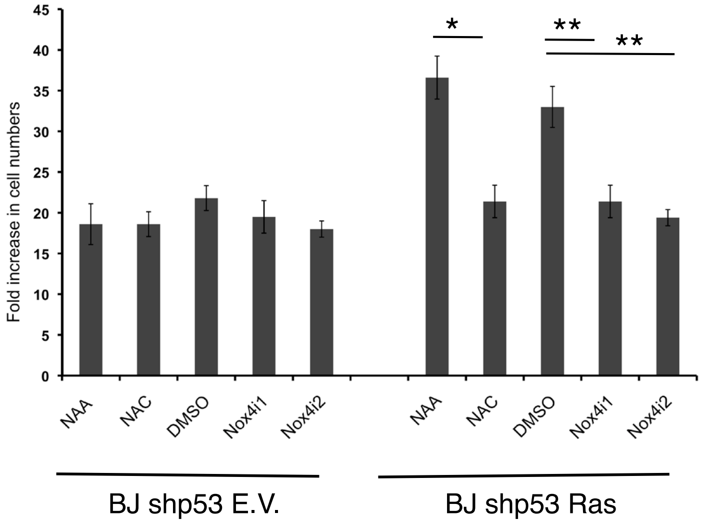

H

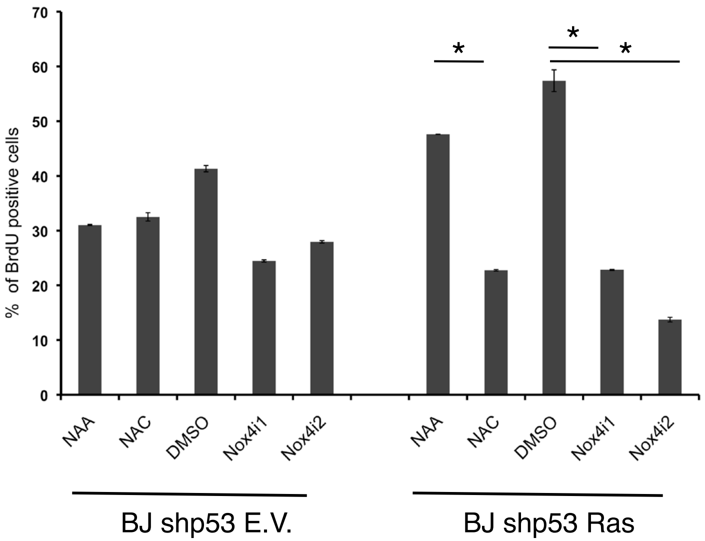

Supplementary 7, Ogrunc et al

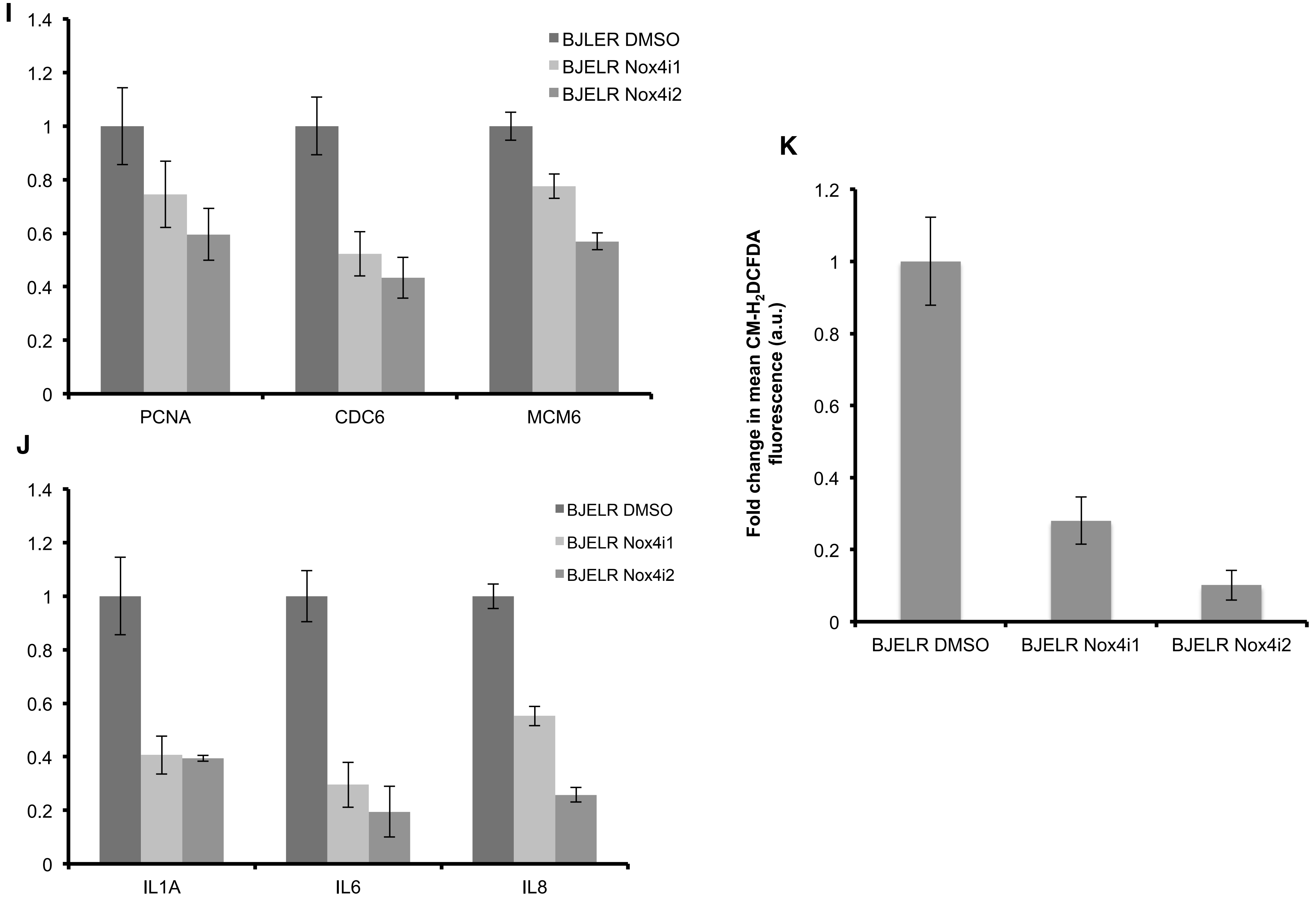

Supplementary 7, Ogrunc et al

L

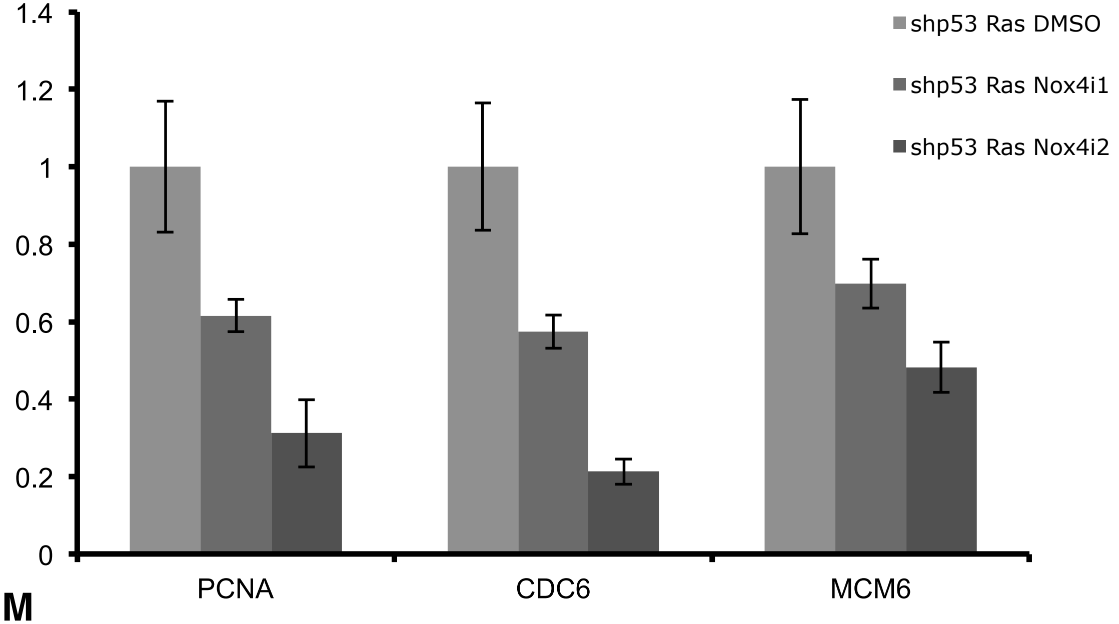

N

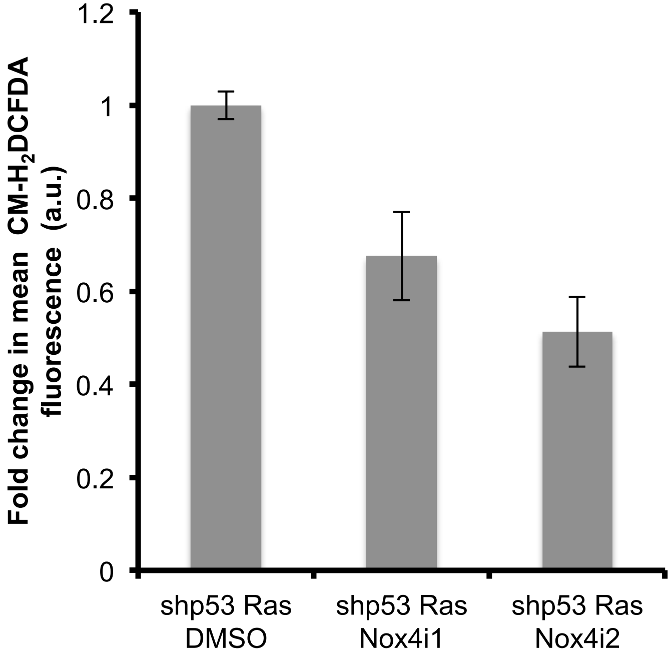

M

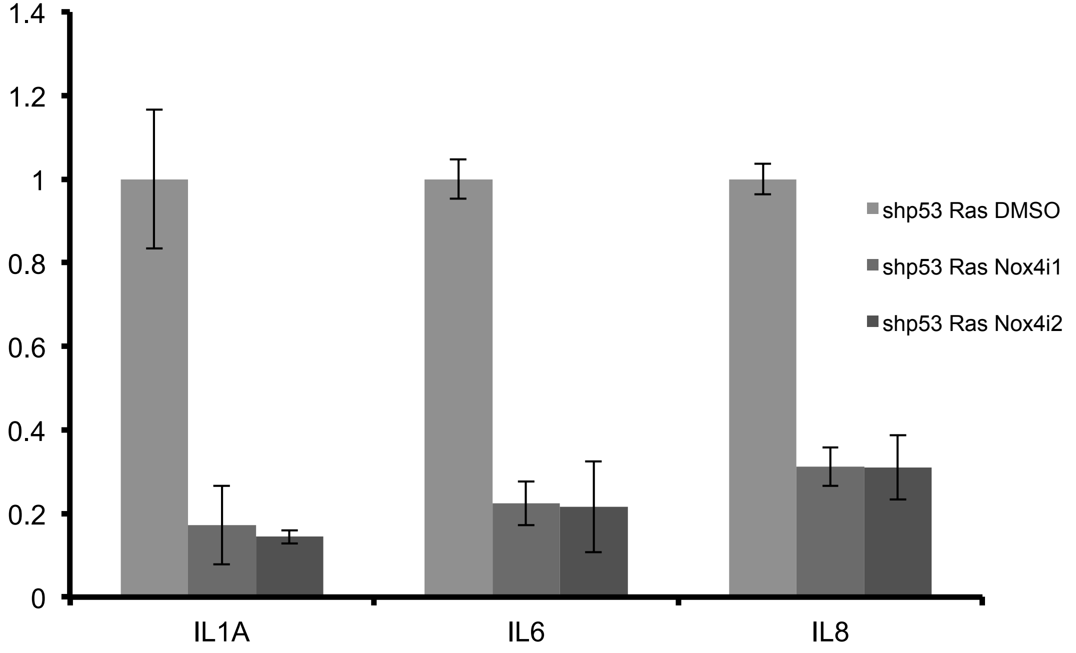

Supplementary 7, Ogrunc et al

O

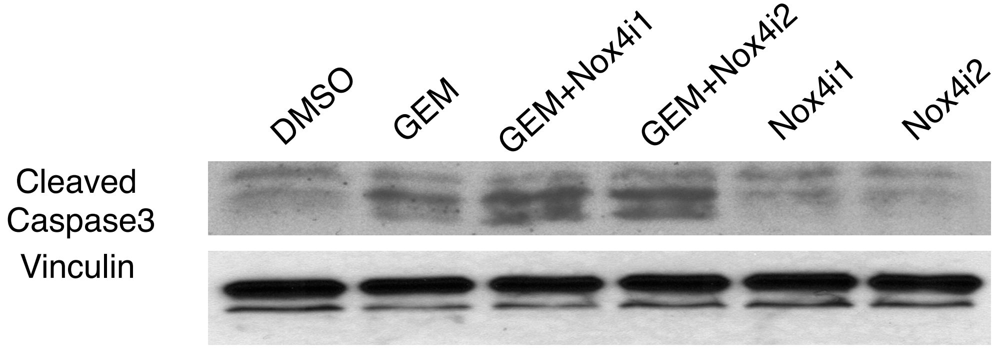

Supplement: Supplementary Information [file cdd201416x1.pdf]
